# Supplementary material for: CDK4/6 Inhibitors Suppress RB-Null Triple-Negative Breast Cancer by Inhibiting Mutant P53 Expression via RBM38 RNA-Binding Protein
Source: Cancers (Basel). 2025 Oct 16;17(20):3339. doi: 10.3390/cancers17203339 (PMC12564691; doi:10.3390/cancers17203339)
Supplement: Supplementary file 1 [file cancers-17-03339-s001.zip › cancers-3927674-supplementary materials/cancers-3927674-supplementary figures.pdf]

Supplemental Figure 1

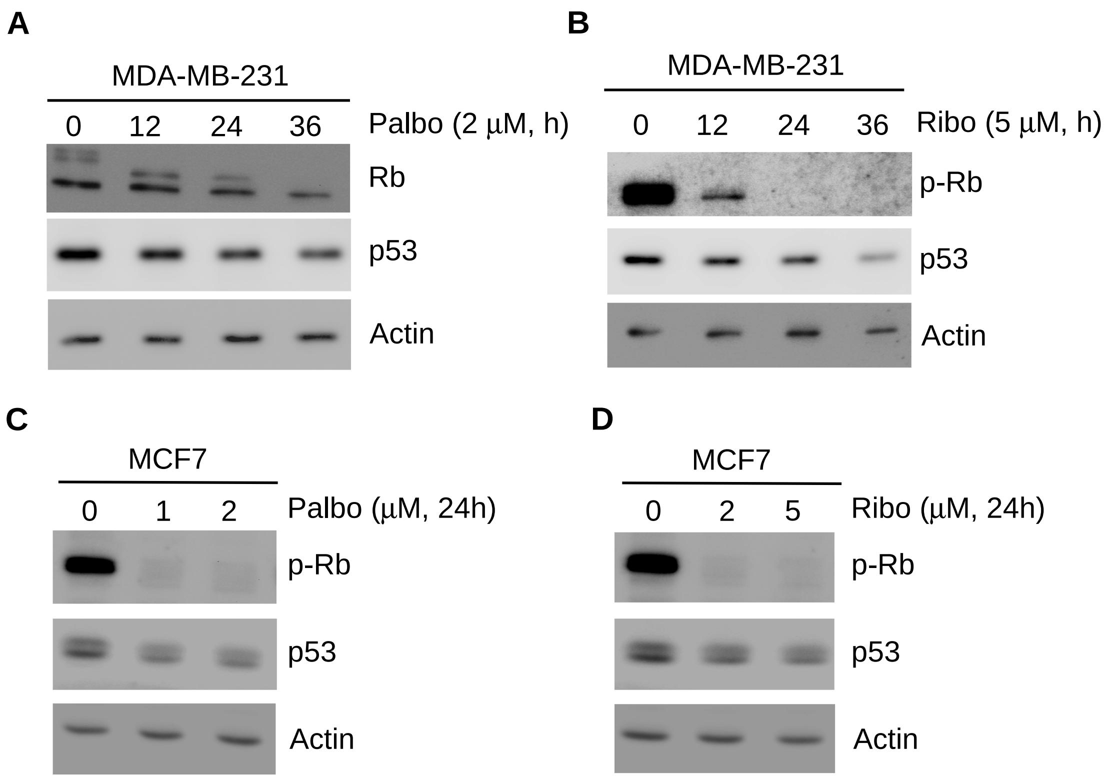

Supplemental Fig. 1 (A) MDA-MB-231 cells were mock-treated or treated with Palbociclib (2 µM) for 0-36 hours, followed by western blot to measure RB, p53 and actin. MDA-MB-231 cells were mock-treated or treated with Ribociclib (5 µM) for 0-36 hours, followed by western blot to measure p-RB, p53 and actin. (C-D) MCF7 cells were mock-treated or treaued with Palbociclib (0-2 µM) (C) or Ribociclib (0-5 µM) (D) for 24 hours, followed by western blot analysis to detect p-Rb, p53 and actin.

Supplemental Figure 2

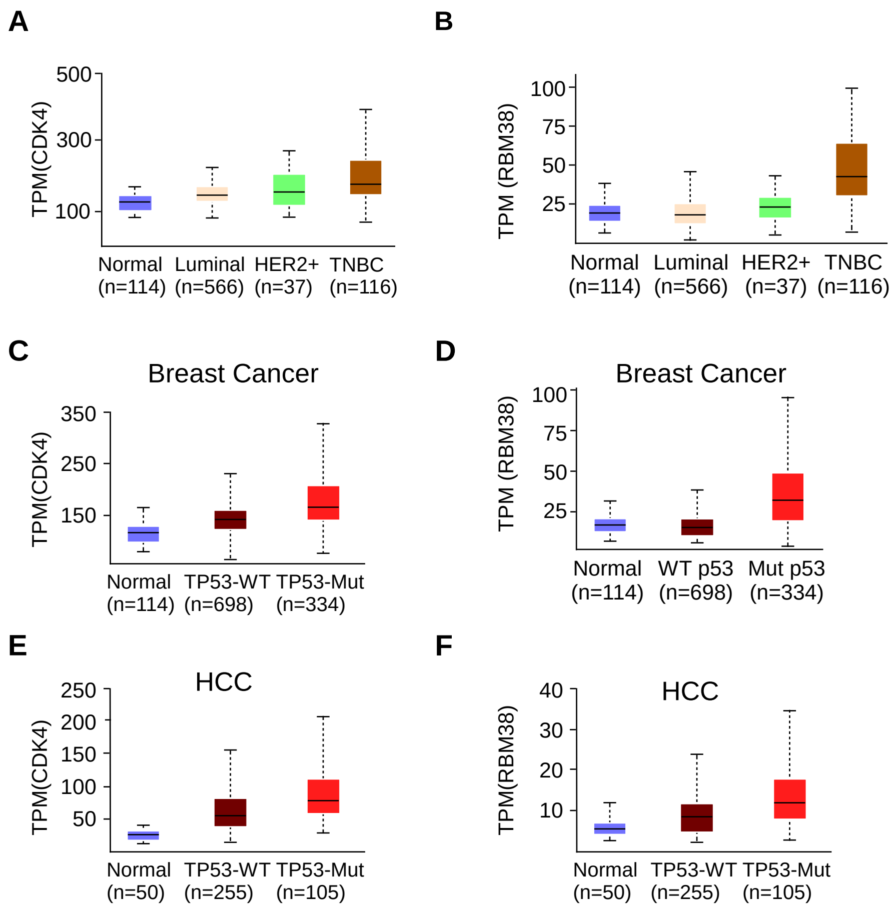

Supplemental Fig. 2 (A-B) The relative level of CDK4 (A) and RBM38 (B) transcripts in normal and various breast cancer tissues. Data are obtained from the UALCAN database. (C-D) The relative level of CDK4 (C) and RBM38 (D) transcripts in normal breast tissue or wild-type p53- or mutant p53-containing breast cancer tissues. (E-F) The relative level of CDK4 (E) and RBM38 (F) transcripts in normal liver tissue or wild-type p53- or mutant p53-containing hepatocellular carcinoma (HCC) tissues. Data are obtained from the UALCAN database.

Supplemental Figure 3

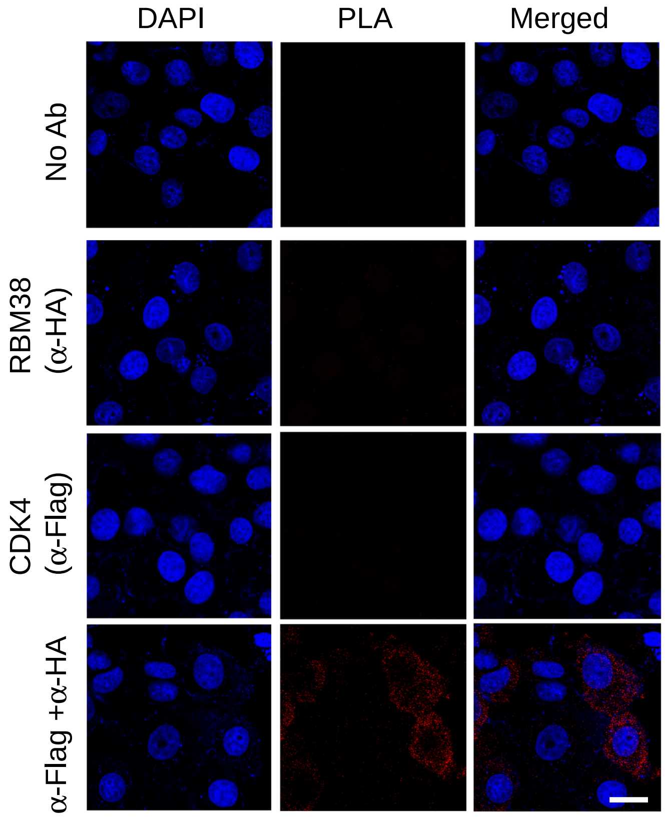

Supplemental Figure 3 MCF7 cells were transiently transfected with Flag-tagged CDK4 and HA-tagged RBM38 for 24 hours, followed by PLA assay as described in "experimental procedures".
