# Supplementary material for: CDK4/6 Inhibitors Suppress RB-Null Triple-Negative Breast Cancer by Inhibiting Mutant P53 Expression via RBM38 RNA-Binding Protein
Source: Cancers (Basel). 2025 Oct 16;17(20):3339. doi: 10.3390/cancers17203339 (PMC12564691; doi:10.3390/cancers17203339)

**Figure 2A**

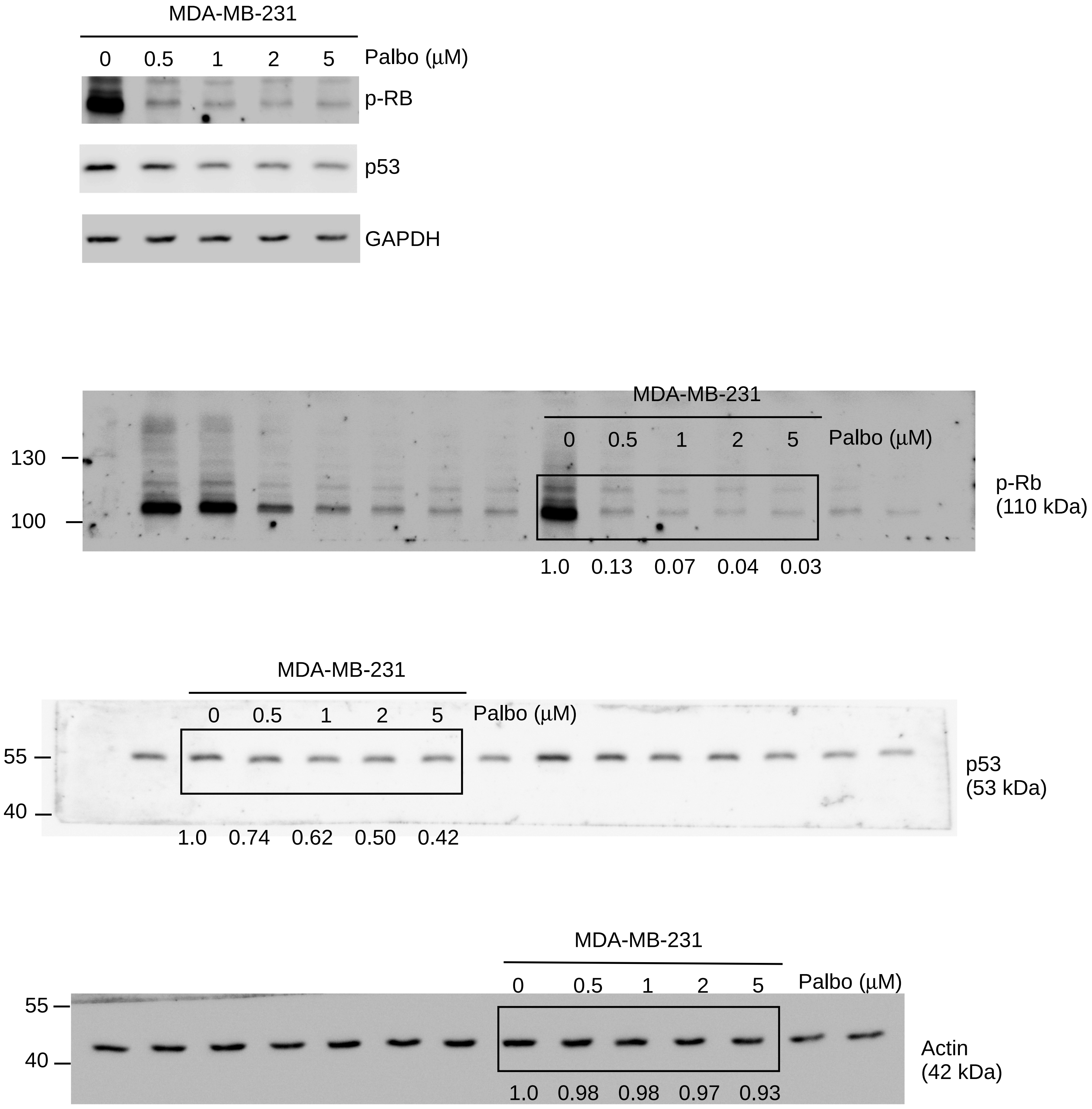
MDA-MB-231
0
0.5
1
2
5
Palbo (μM)
p-RB
p53
GAPDH
MDA-MB-231
0
0.5
1
2
5
Palbo (μM)
130
100
p-Rb
(110 kDa)
1.0
0.13
0.07
0.04
0.03
MDA-MB-231
0
0.5
1
2
5
Palbo (μM)
55
40
p53
(53 kDa)
1.0
0.74
0.62
0.50
0.42
MDA-MB-231
0
0.5
1
2
5
Palbo (μM)
55
40
Actin
(42 kDa)
1.0
0.98
0.98
0.97
0.93

**Figure 2B**

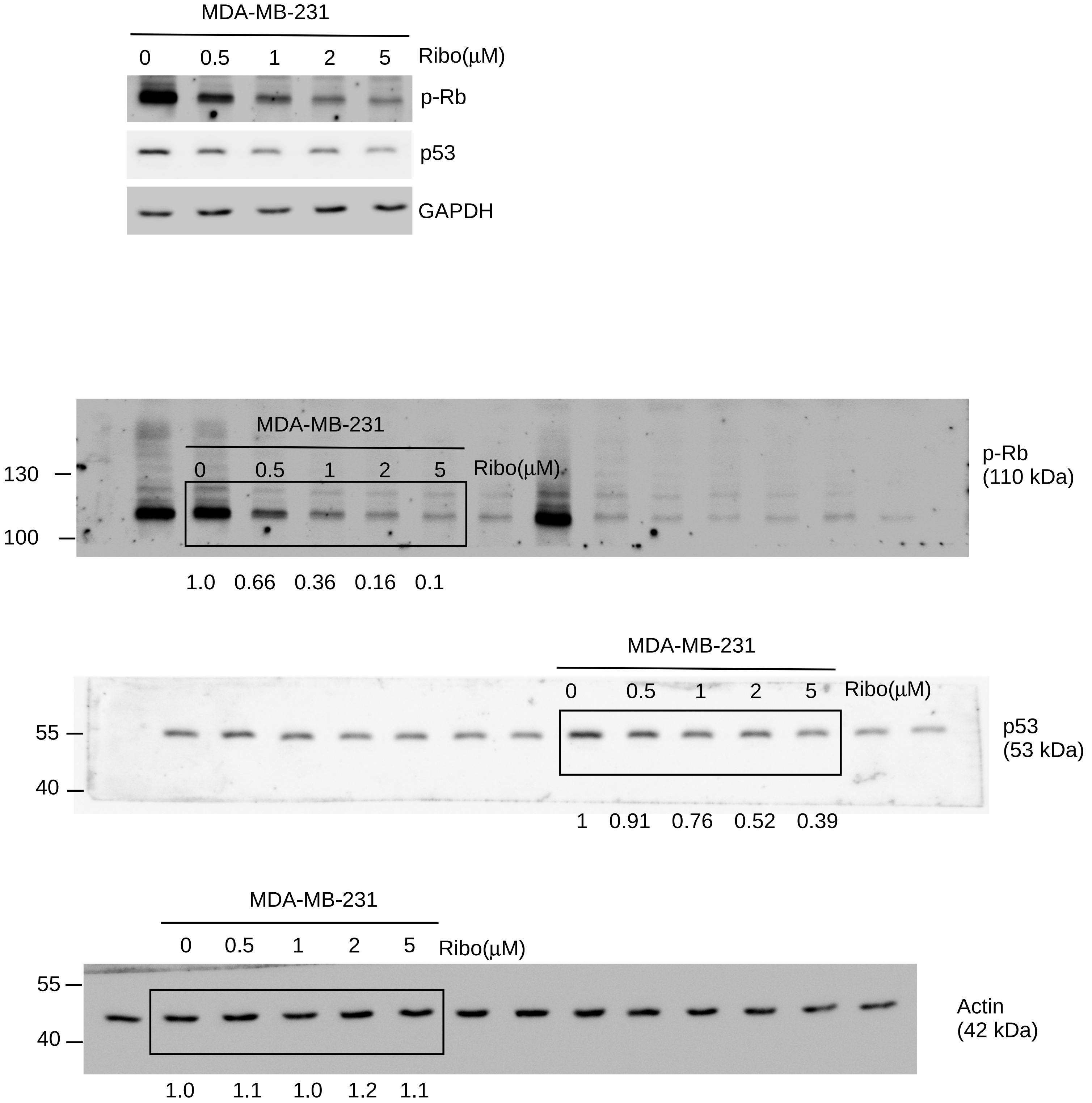
MDA-MB-231
0 0.5 1 2 5 Ribo(μM)
p-Rb
p53
GAPDH
MDA-MB-231
0 0.5 1 2 5 Ribo(μM)
130
100
p-Rb (110 kDa)
1.0 0.66 0.36 0.16 0.1
MDA-MB-231
0 0.5 1 2 5 Ribo(μM)
55
40
p53 (53 kDa)
1 0.91 0.76 0.52 0.39
MDA-MB-231
0 0.5 1 2 5 Ribo(μM)
55
40
Actin (42 kDa)
1.0 1.1 1.0 1.2 1.1

**Figure 2C**

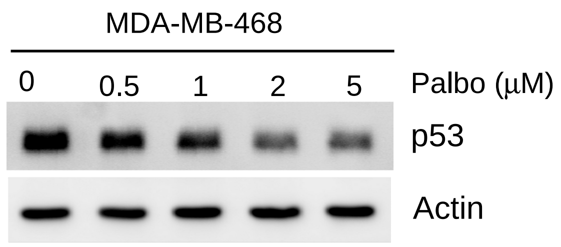
MDA-MB-468
0
0.5
1
2
5
Palbo (μM)
p53
Actin

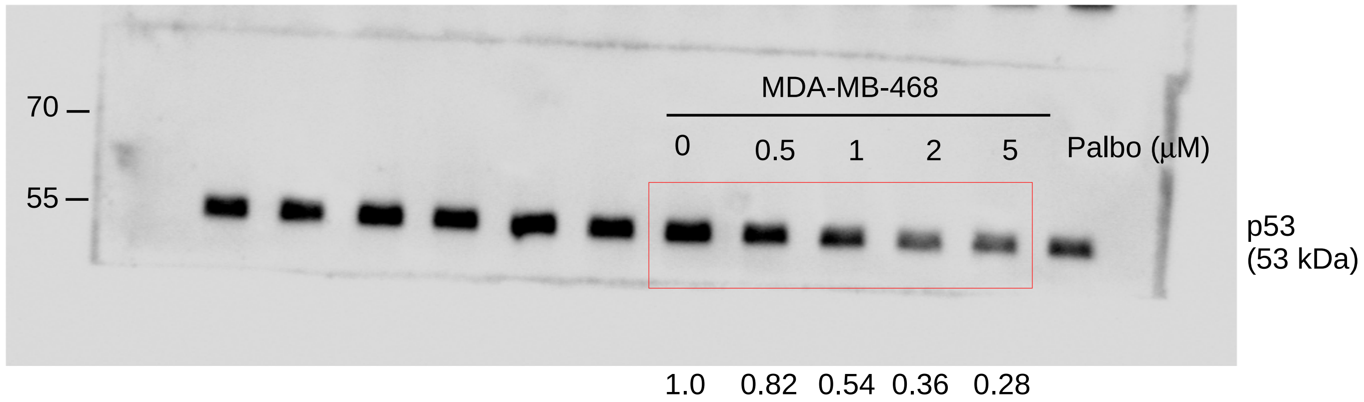
70
55
MDA-MB-468
0
0.5
1
2
5
Palbo (μM)
p53
(53 kDa)
1.0
0.82
0.54
0.36
0.28

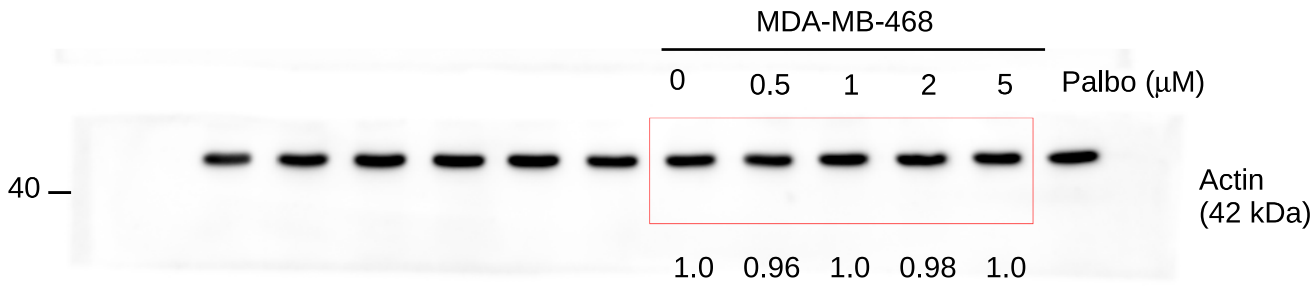
MDA-MB-468
0
0.5
1
2
5
Palbo (μM)
40
Actin
(42 kDa)
1.0
0.96
1.0
0.98
1.0

**Figure 2D**

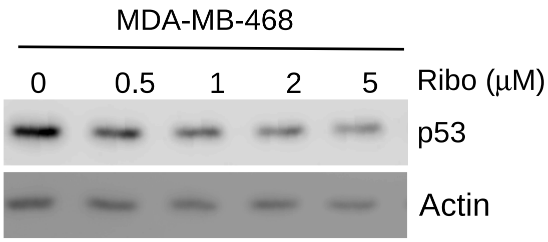


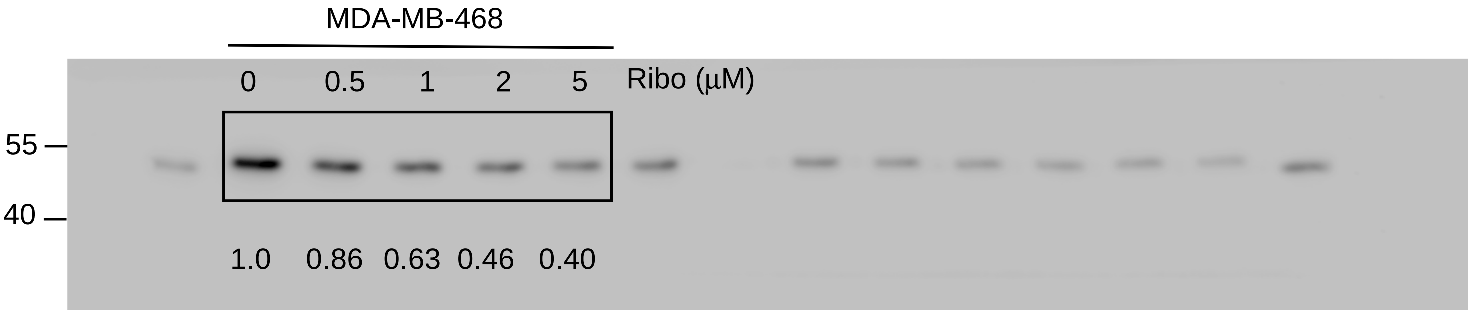


p53 (53 kDa)

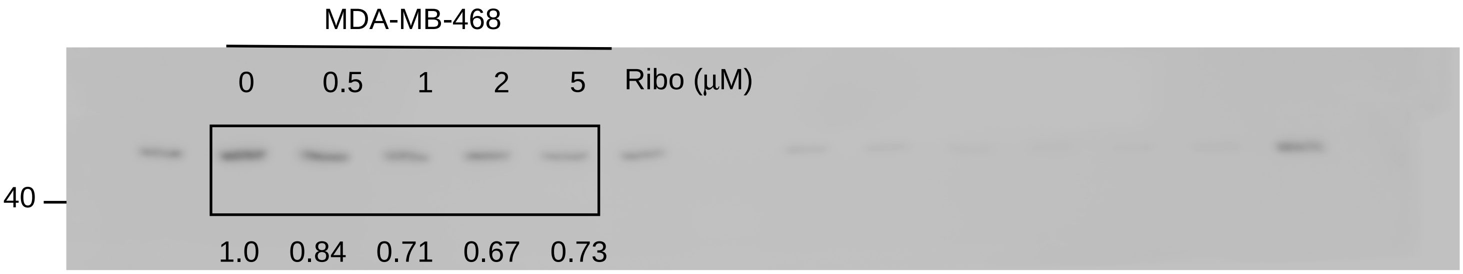


Actin (42 kDa)

**Figure 2E**

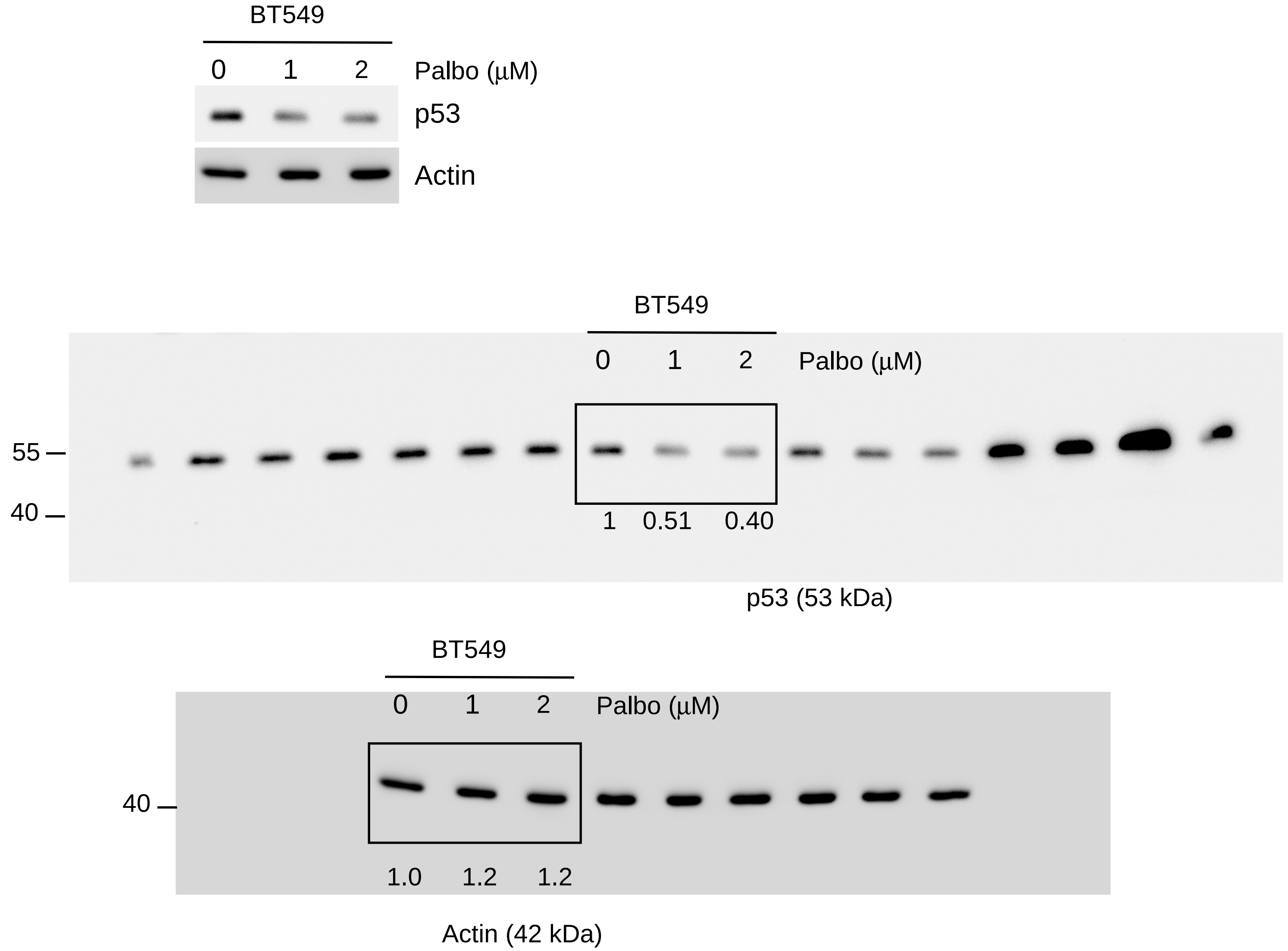
BT549
0 1 2 Palbo (μM)
p53
Actin
BT549
0 1 2 Palbo (μM)
55
40
1 0.51 0.40
p53 (53 kDa)
BT549
0 1 2 Palbo (μM)
40
1.0 1.2 1.2
Actin (42 kDa)

**Figure 2F**

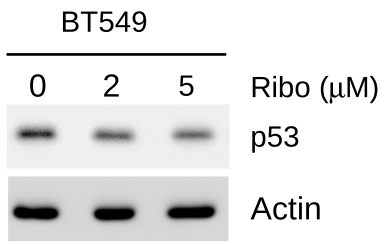


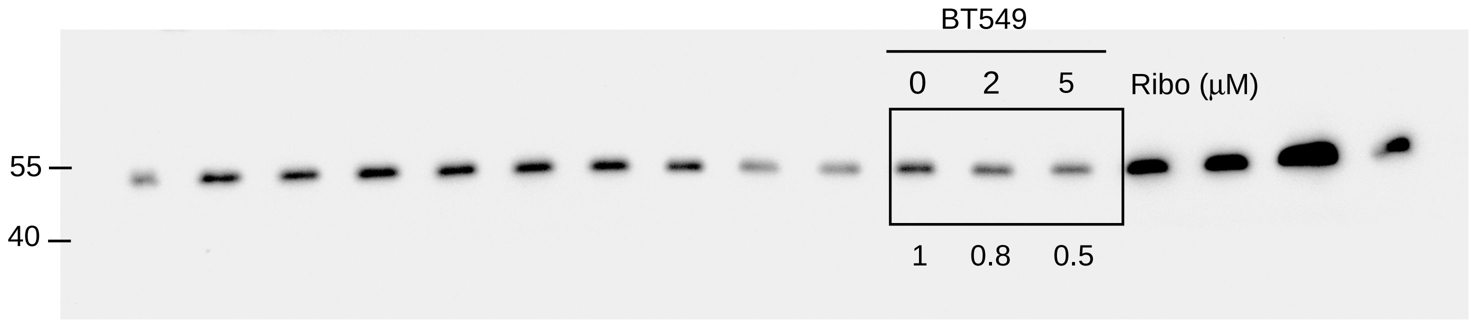


p53 (53 kDa)

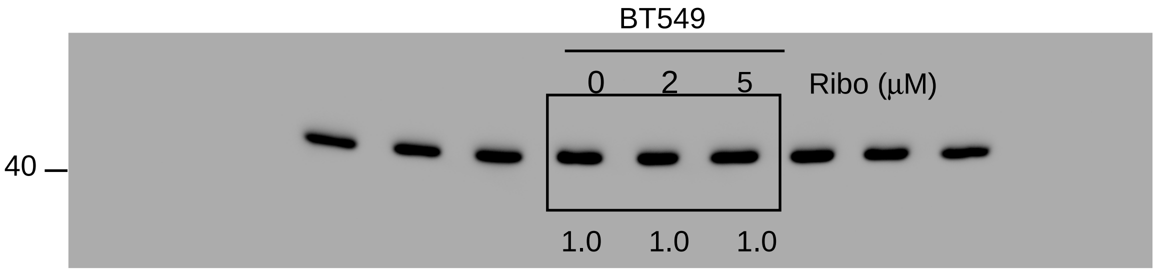


Actin (42 kDa)

**Figure 2G**

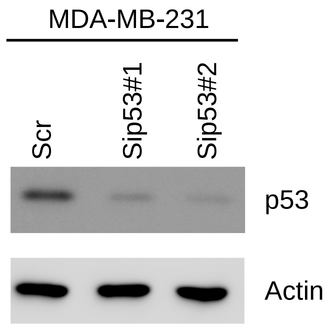


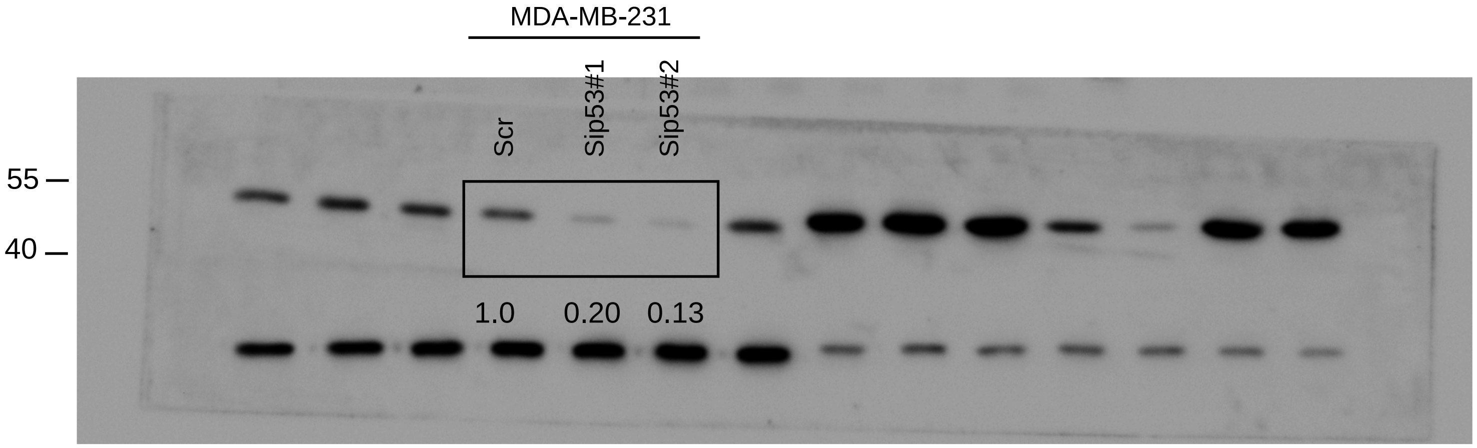


p53 (5 kDa)

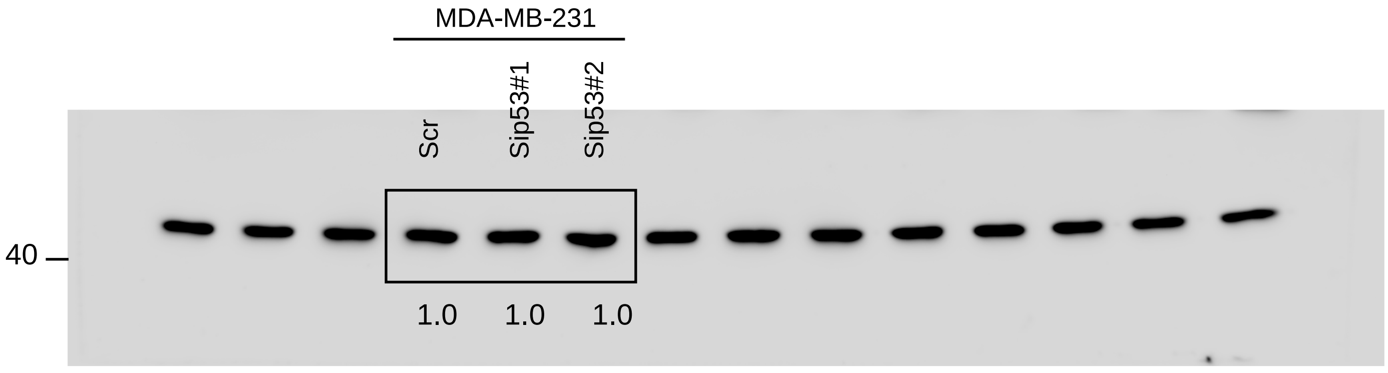


Actin (42 kDa)

**Figure 2I**

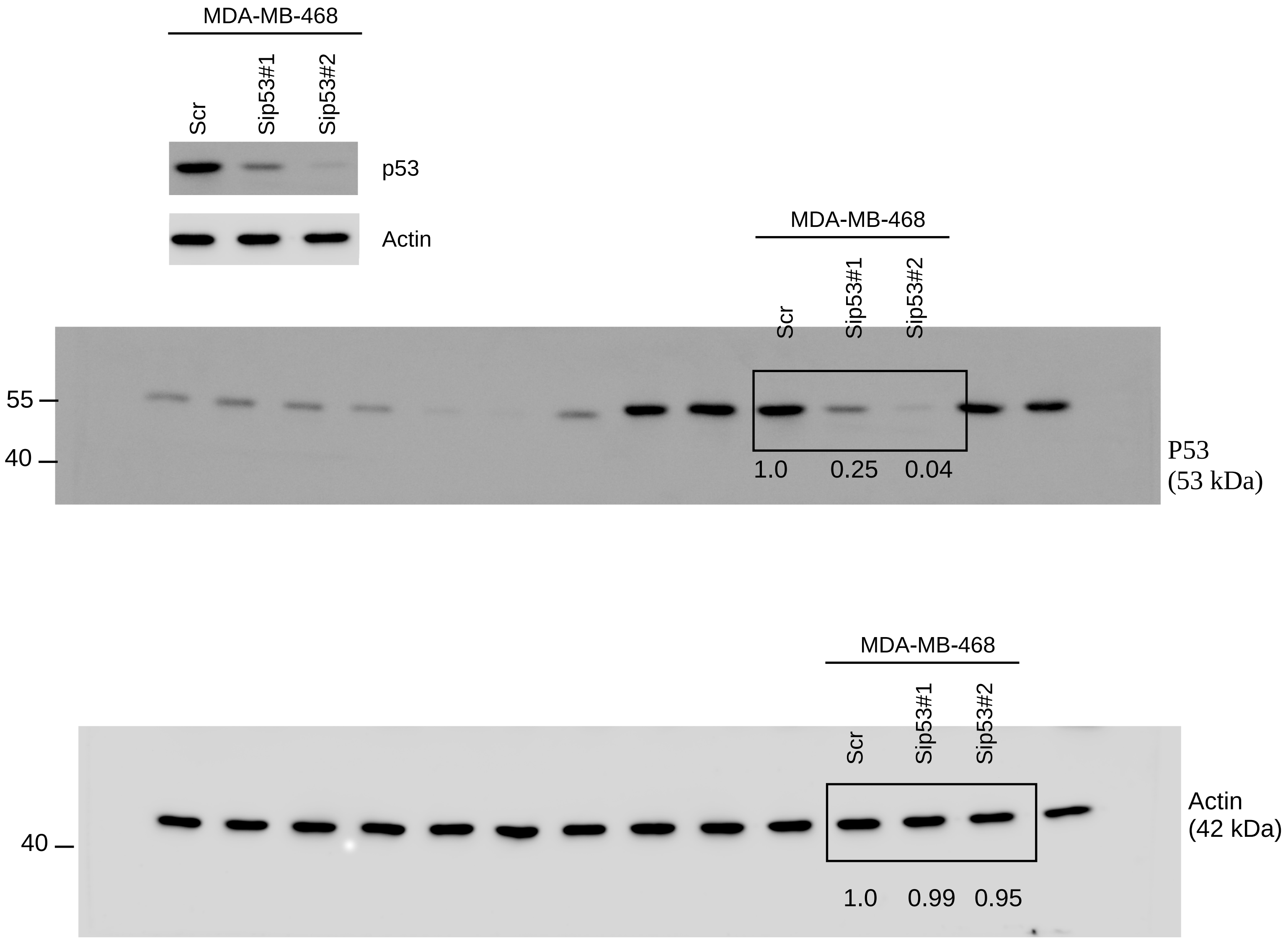

MDA-MB-468
Scr
Sip53#1
Sip53#2
p53
Actin
MDA-MB-468
Scr
Sip53#1
Sip53#2
55
40
1.0
0.25
0.04
P53
(53 kDa)
MDA-MB-468
Scr
Sip53#1
Sip53#2
40
Actin
(42 kDa)
1.0
0.99
0.95

Figure 3A

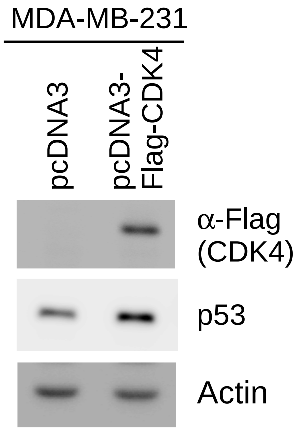

MDA-MB-231
pcDNA3
pcDNA3-Flag-CDK4
α-Flag (CDK4)
p53
Actin


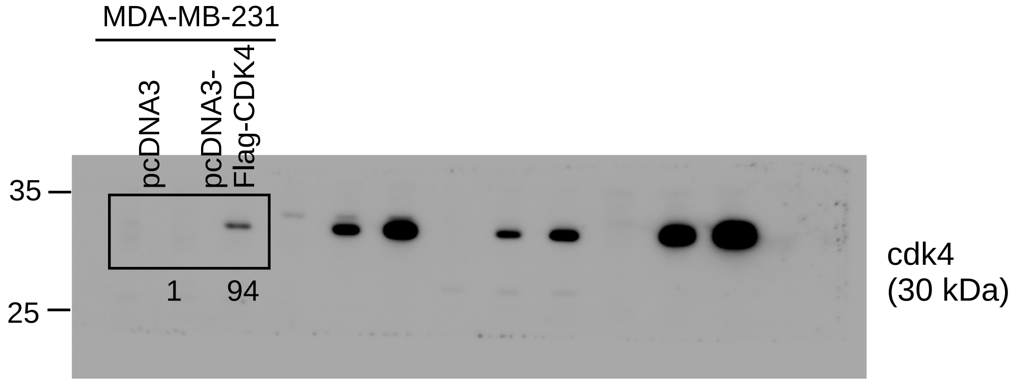

MDA-MB-231
pcDNA3
pcDNA3-Flag-CDK4
35
25
1
94
cdk4 (30 kDa)


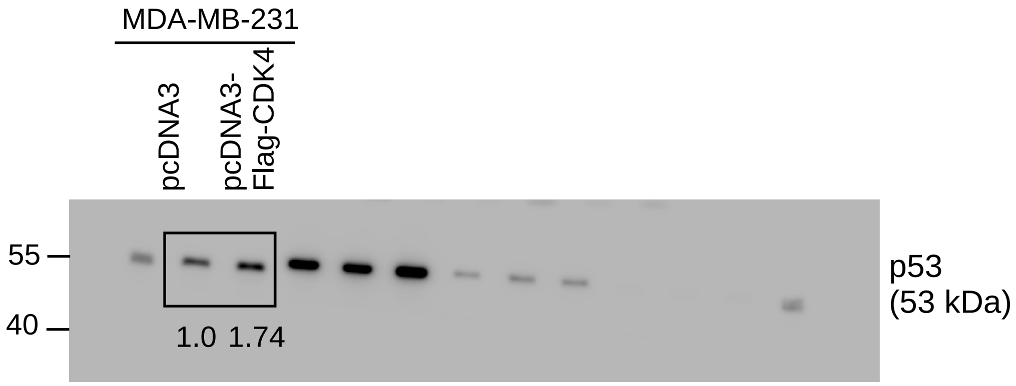

MDA-MB-231
pcDNA3
pcDNA3-Flag-CDK4
55
40
1.0
1.74
p53 (53 kDa)


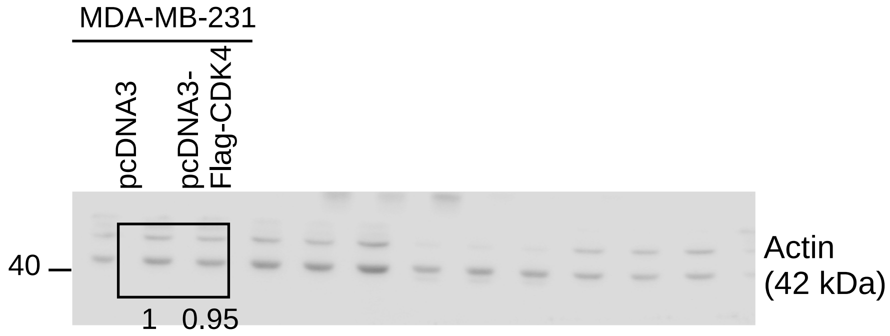

MDA-MB-231
pcDNA3
pcDNA3-Flag-CDK4
40
1
0.95
Actin (42 kDa)

**Figure 3B**

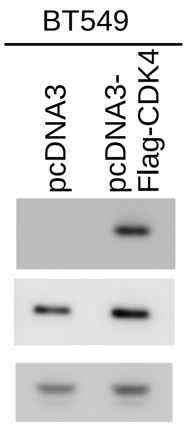

BT549
pcDNA3
pcDNA3-Flag-CDK4


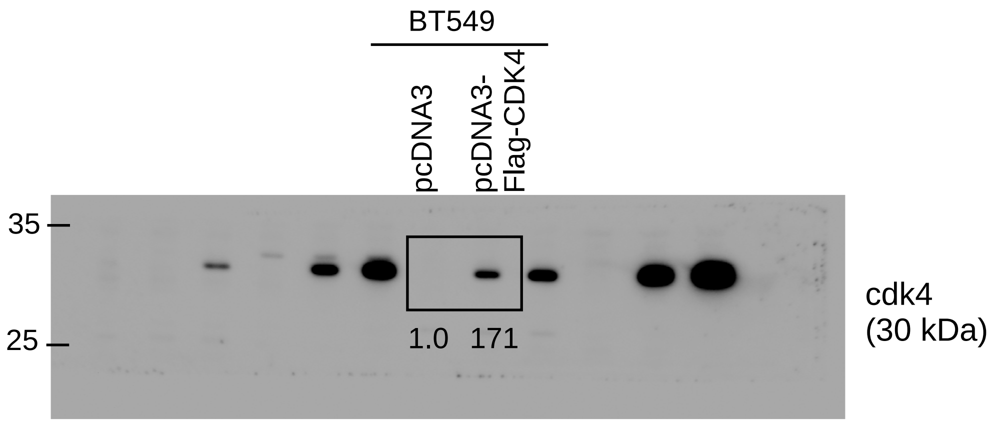

BT549
pcDNA3
pcDNA3-Flag-CDK4
35
25
1.0
171
cdk4
(30 kDa)


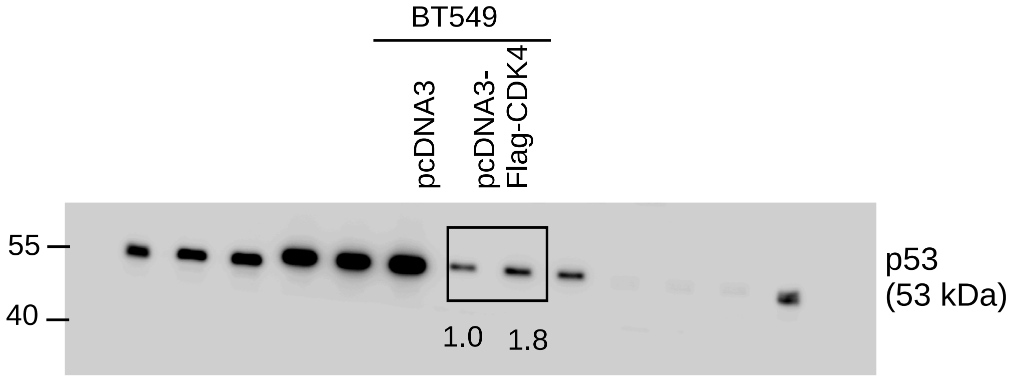

BT549
pcDNA3
pcDNA3-Flag-CDK4
55
40
1.0
1.8
p53
(53 kDa)


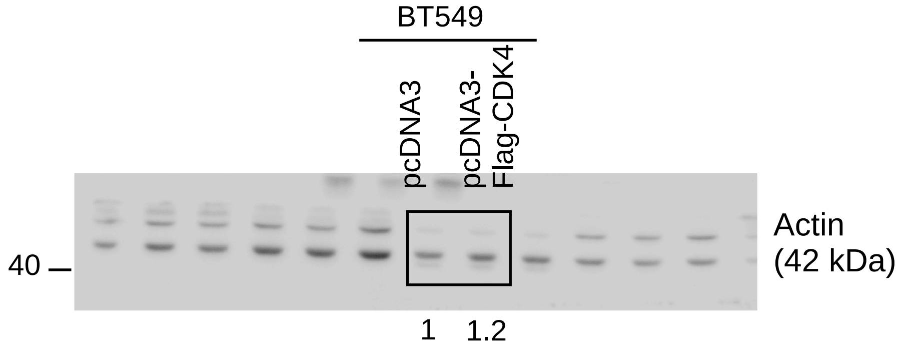

BT549
pcDNA3
pcDNA3-Flag-CDK4
40
1
1.2
Actin
(42 kDa)

**Figure 3C**

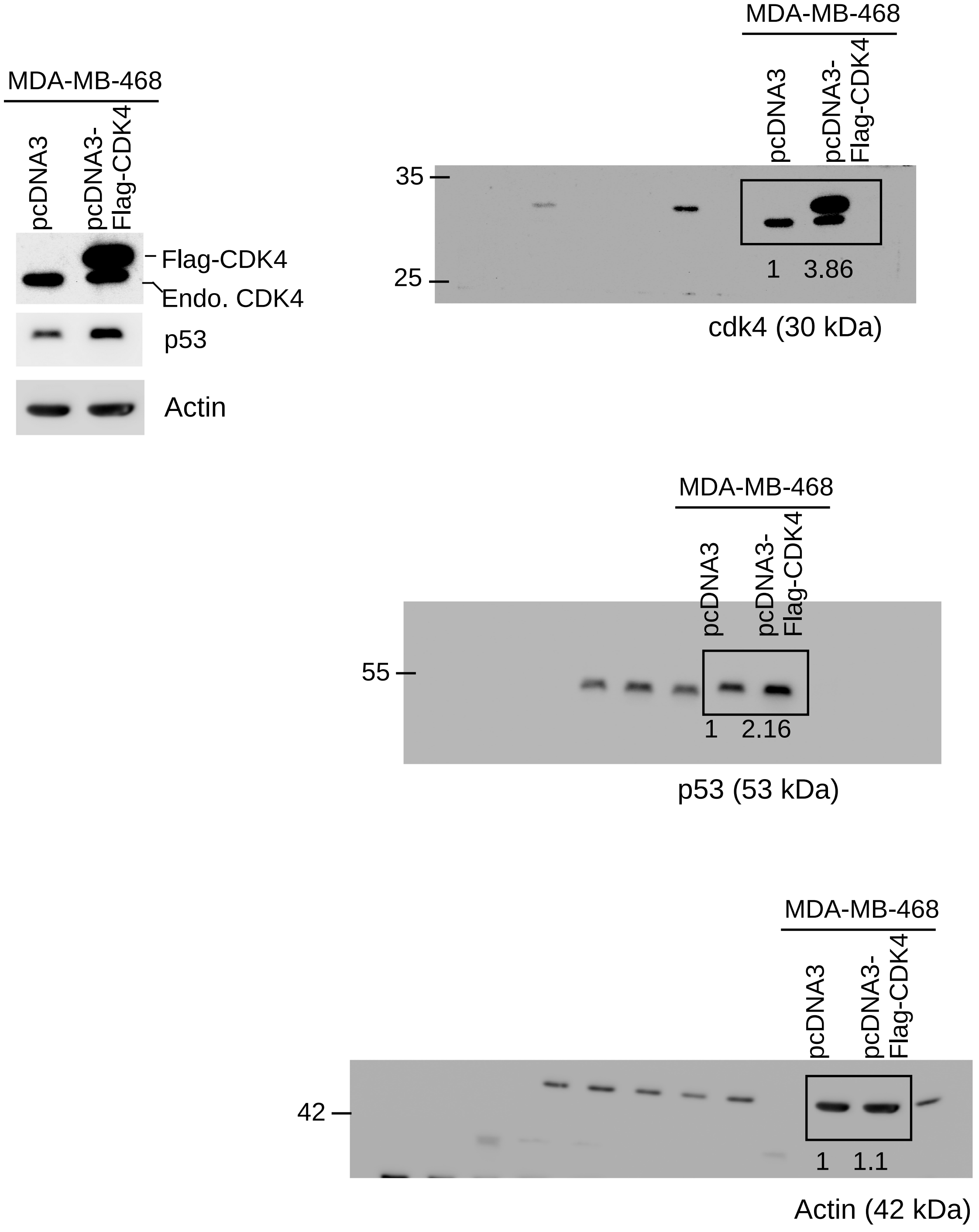

MDA-MB-468
pcDNA3
pcDNA3-Flag-CDK4
Flag-CDK4
Endo. CDK4
p53
Actin
MDA-MB-468
pcDNA3
pcDNA3-Flag-CDK4
35
25
1
3.86
cdk4 (30 kDa)
MDA-MB-468
pcDNA3
pcDNA3-Flag-CDK4
55
1
2.16
p53 (53 kDa)
MDA-MB-468
pcDNA3
pcDNA3-Flag-CDK4
42
1
1.1
Actin (42 kDa)

**Figure 3D**

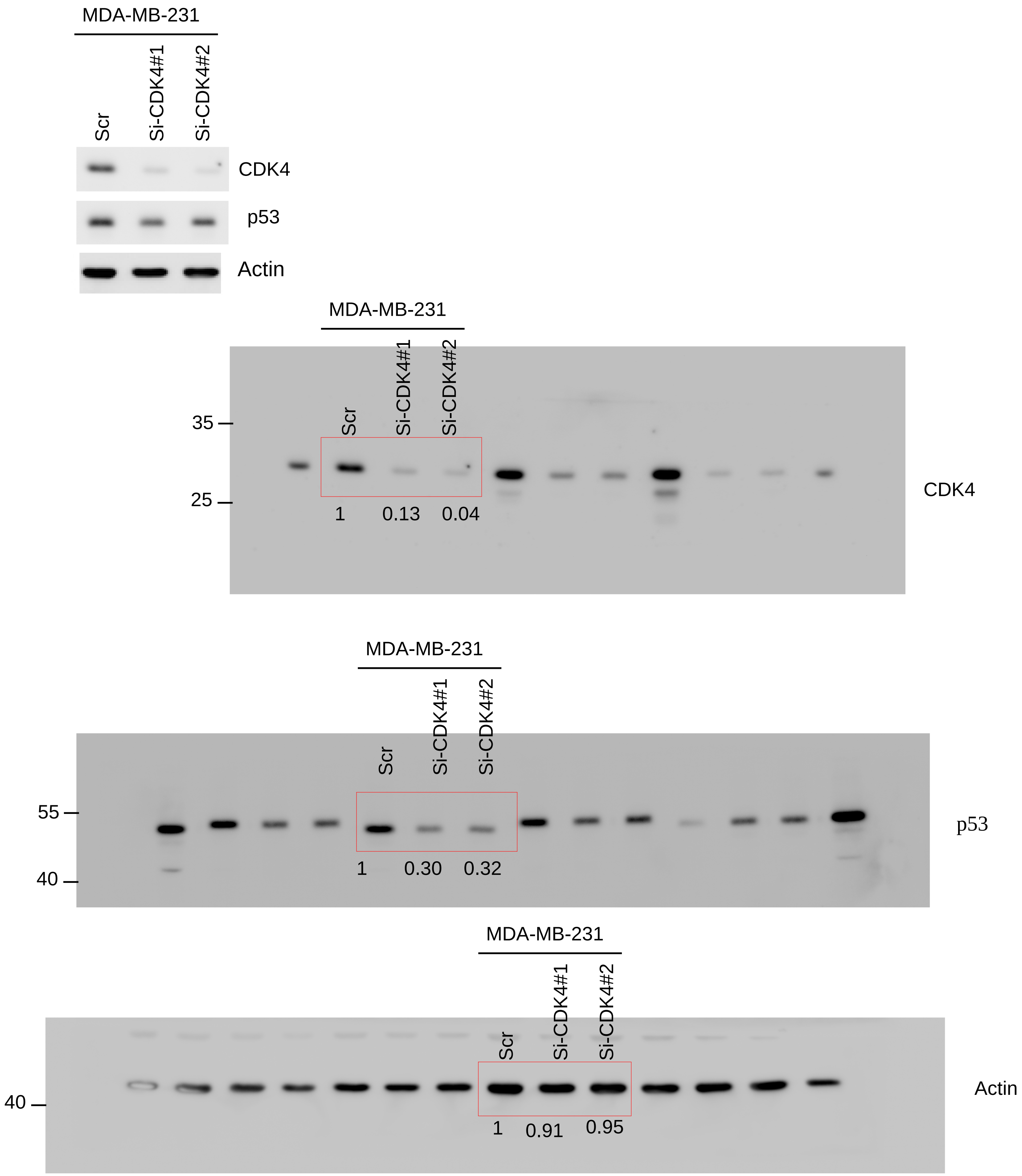
MDA-MB-231
Scr
Si-CDK4#1
Si-CDK4#2
CDK4
p53
Actin
MDA-MB-231
Scr
Si-CDK4#1
Si-CDK4#2
35
25
1
0.13
0.04
CDK4
MDA-MB-231
Scr
Si-CDK4#1
Si-CDK4#2
55
40
1
0.30
0.32
p53
MDA-MB-231
Scr
Si-CDK4#1
Si-CDK4#2
40
1
0.91
0.95
Actin

**Figure 3E**

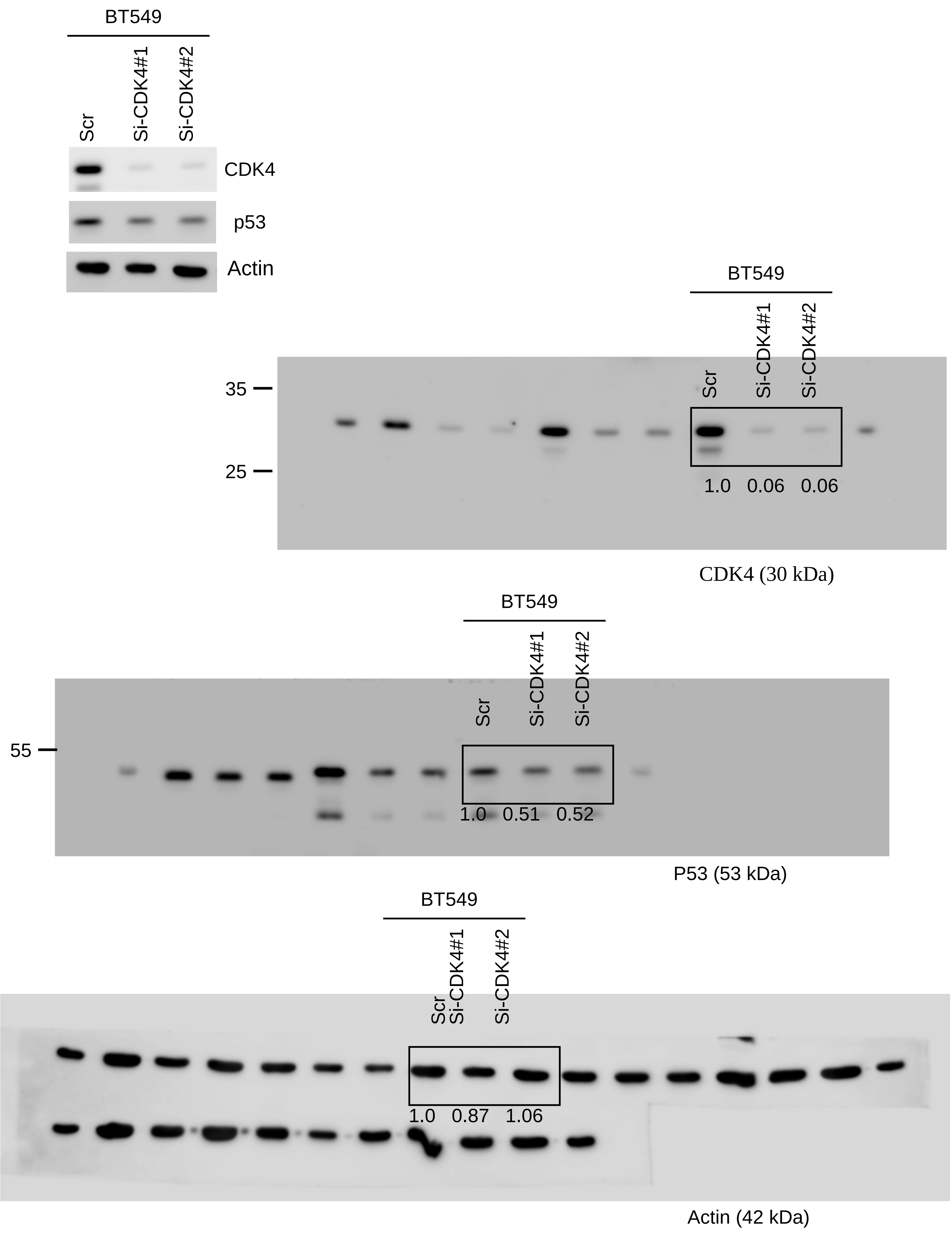

BT549
Scr
Si-CDK4#1
Si-CDK4#2
CDK4
p53
Actin
BT549
Scr
Si-CDK4#1
Si-CDK4#2
35
25
1.0
0.06
0.06
CDK4 (30 kDa)
BT549
Scr
Si-CDK4#1
Si-CDK4#2
55
1.0
0.51
0.52
P53 (53 kDa)
BT549
Scr
Si-CDK4#1
Si-CDK4#2
40
1.0
0.87
1.06
Actin (42 kDa)

**Figure 3F**

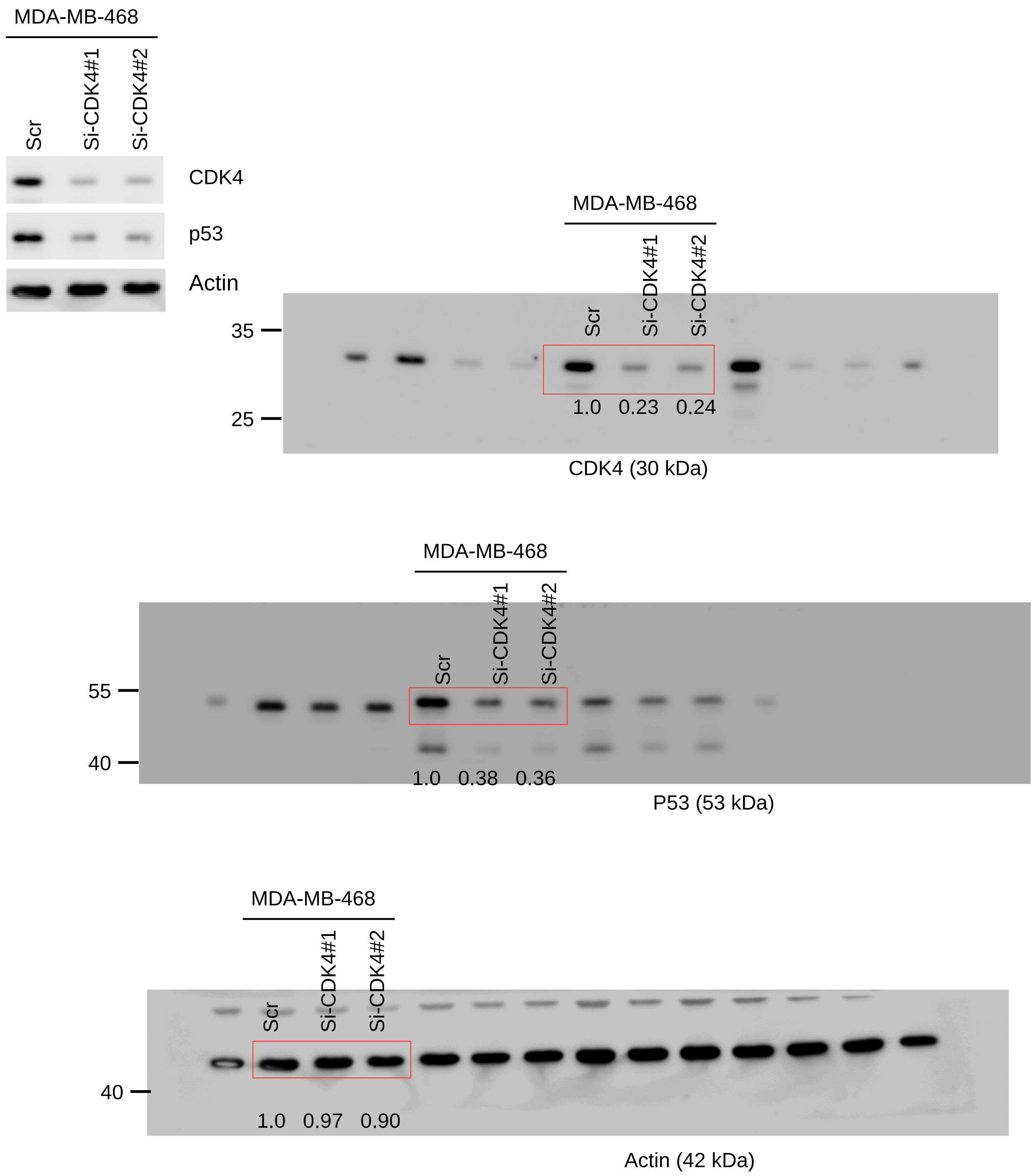
MDA-MB-468
Scr
Si-CDK4#1
Si-CDK4#2
CDK4
p53
Actin
MDA-MB-468
Scr
Si-CDK4#1
Si-CDK4#2
35
25
1.0 0.23 0.24
CDK4 (30 kDa)
MDA-MB-468
Scr
Si-CDK4#1
Si-CDK4#2
55
40
1.0 0.38 0.36
P53 (53 kDa)
MDA-MB-468
Scr
Si-CDK4#1
Si-CDK4#2
40
1.0 0.97 0.90
Actin (42 kDa)

**Figure 3G**

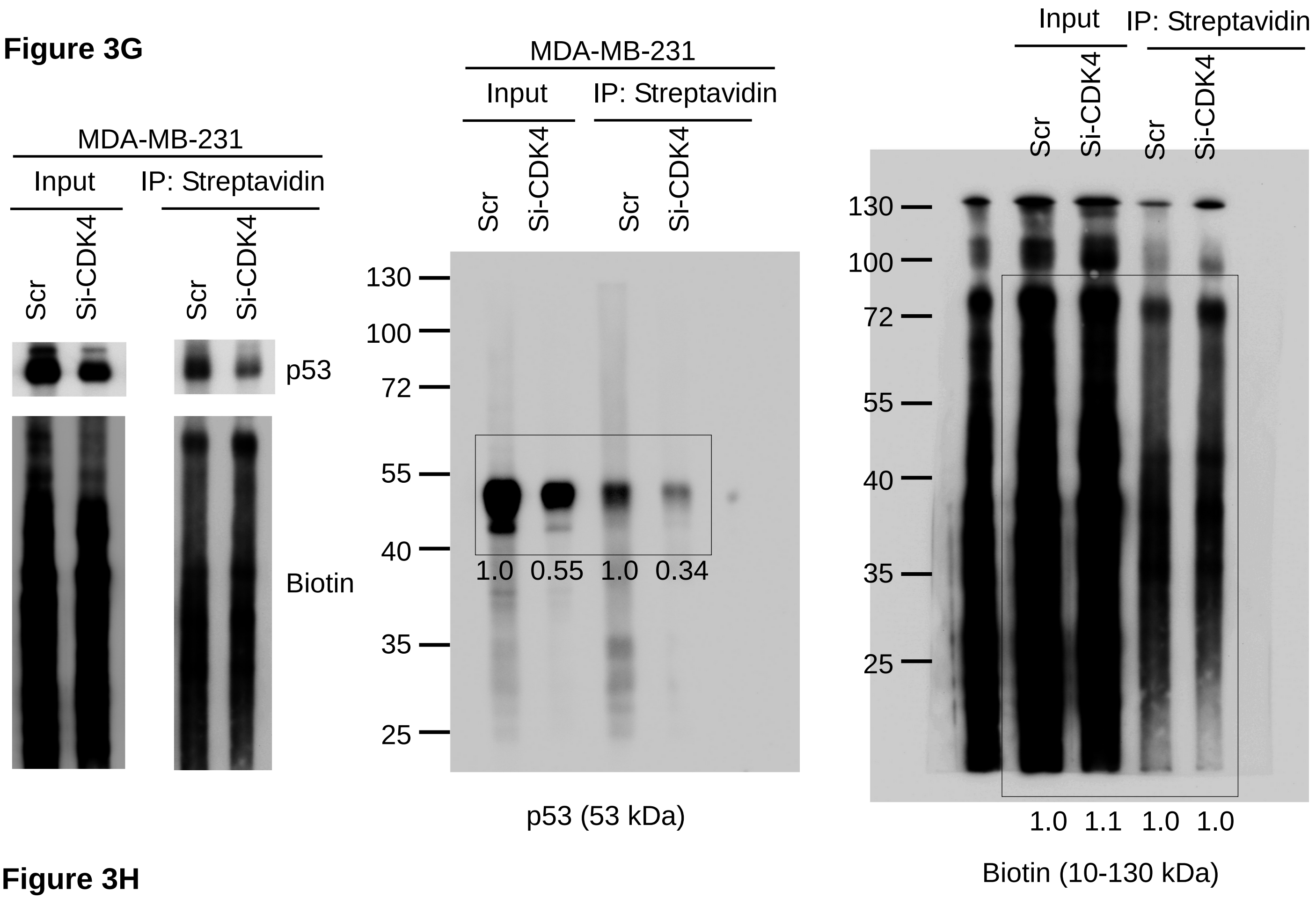


**Figure 3H**

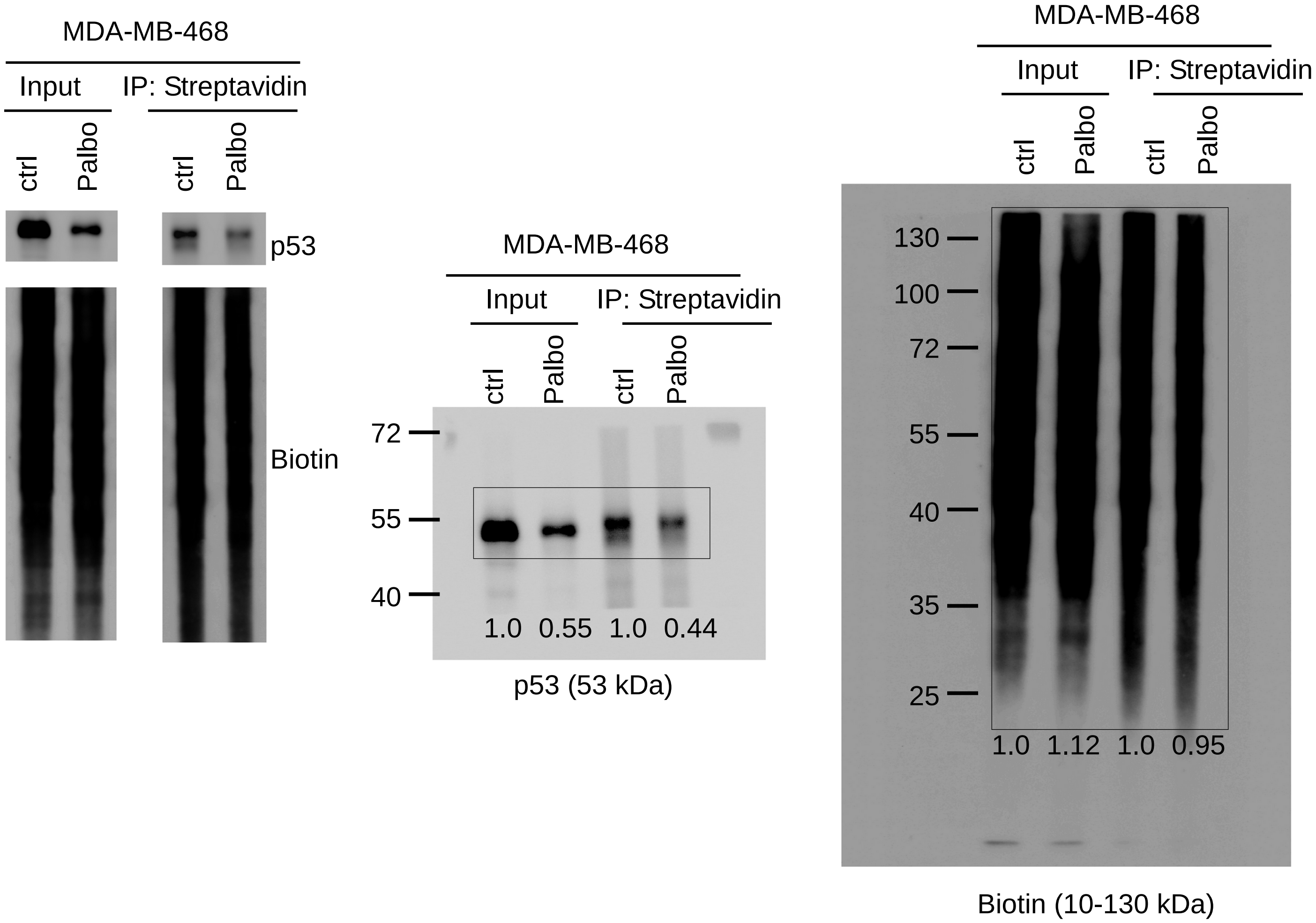

**Figure 3I**

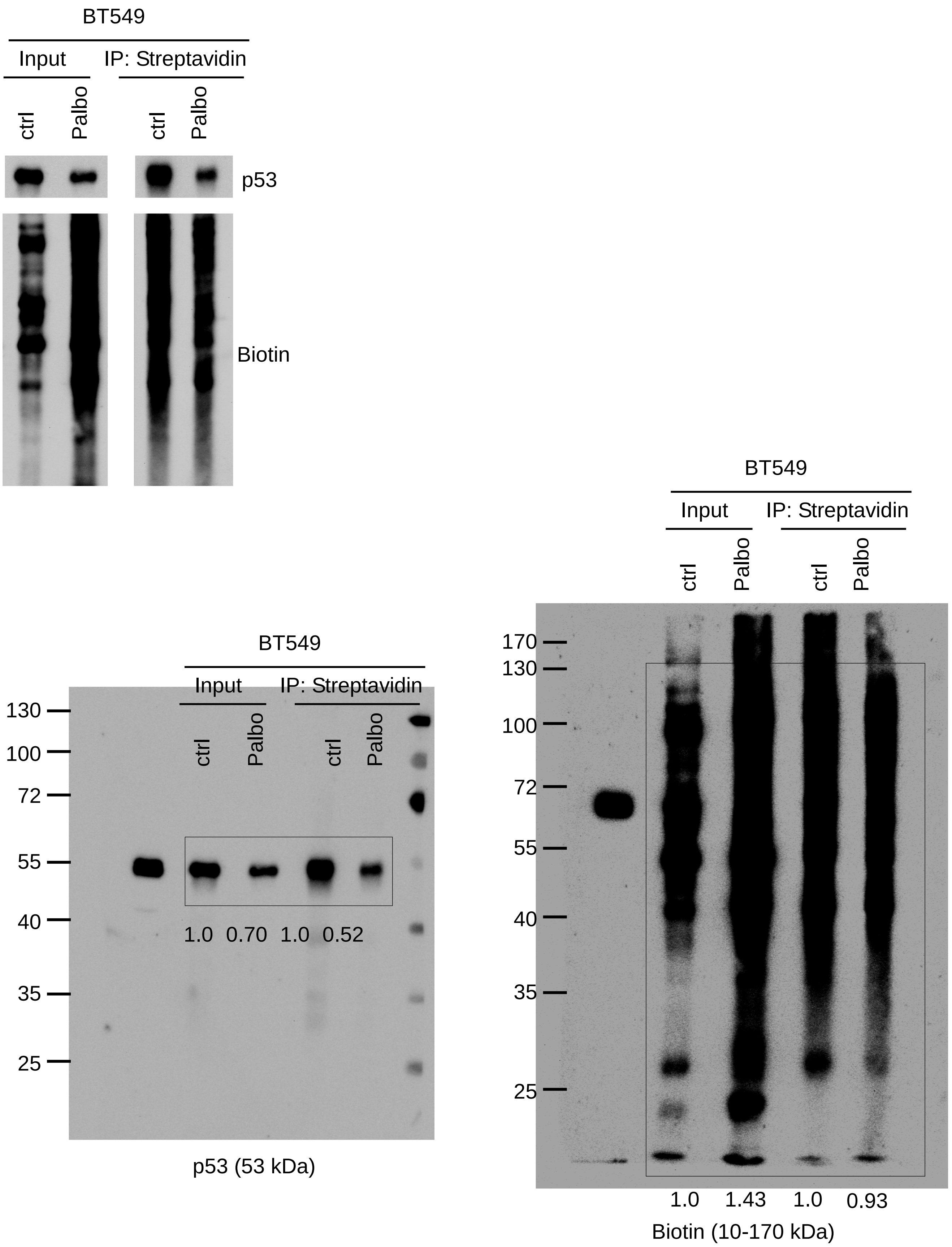
BT549
Input
IP: Streptavidin
ctrl
Palbo
ctrl
Palbo
p53
Biotin
BT549
Input
IP: Streptavidin
ctrl
Palbo
ctrl
Palbo
130
100
72
55
40
35
25
1.0 0.70 1.0 0.52
p53 (53 kDa)
BT549
Input
IP: Streptavidin
ctrl
Palbo
ctrl
Palbo
170
130
100
72
55
40
35
25
1.0 1.43 1.0 0.93
Biotin (10-170 kDa)

**Figure 4A**

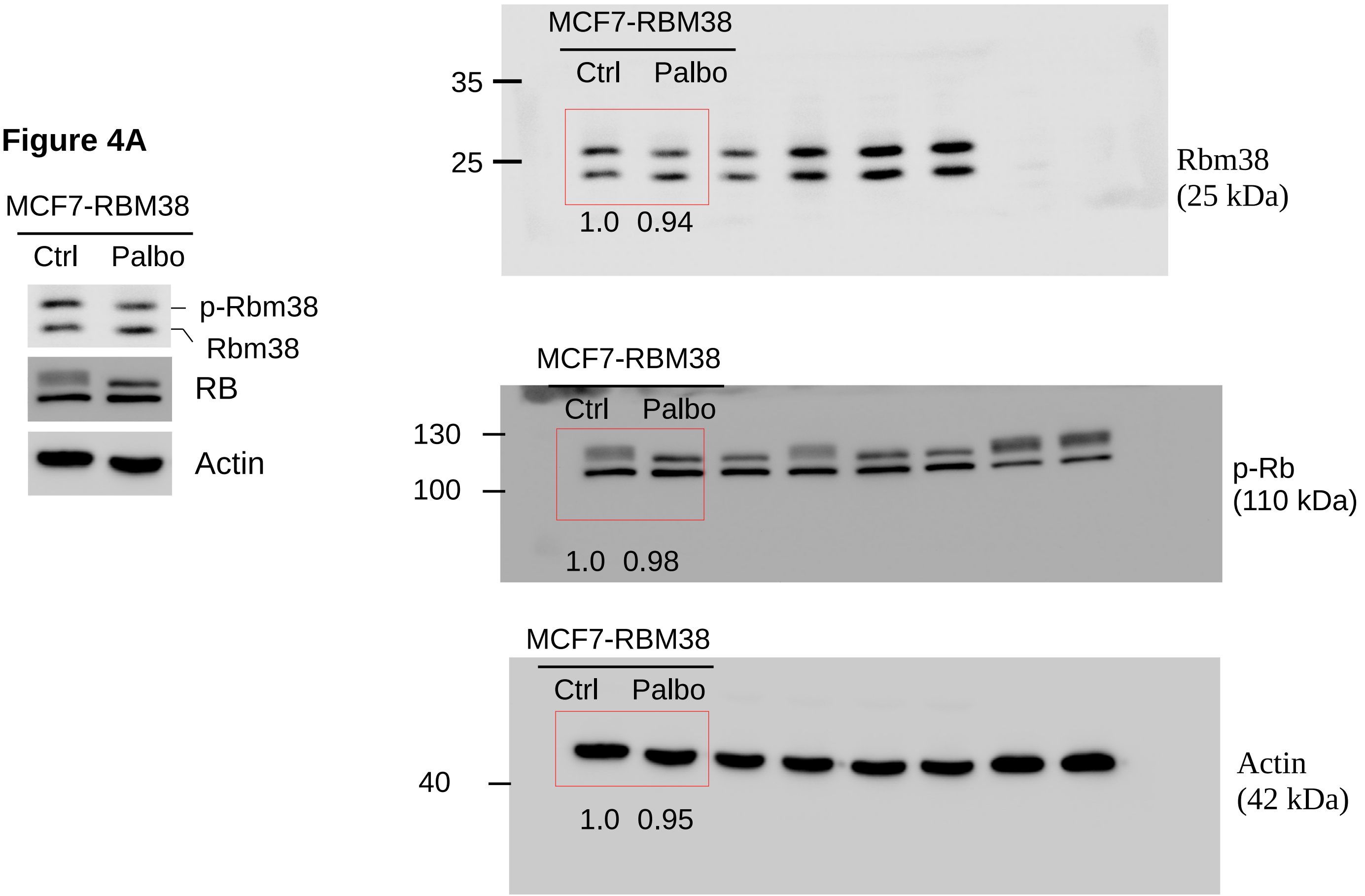


**Figure 4B**

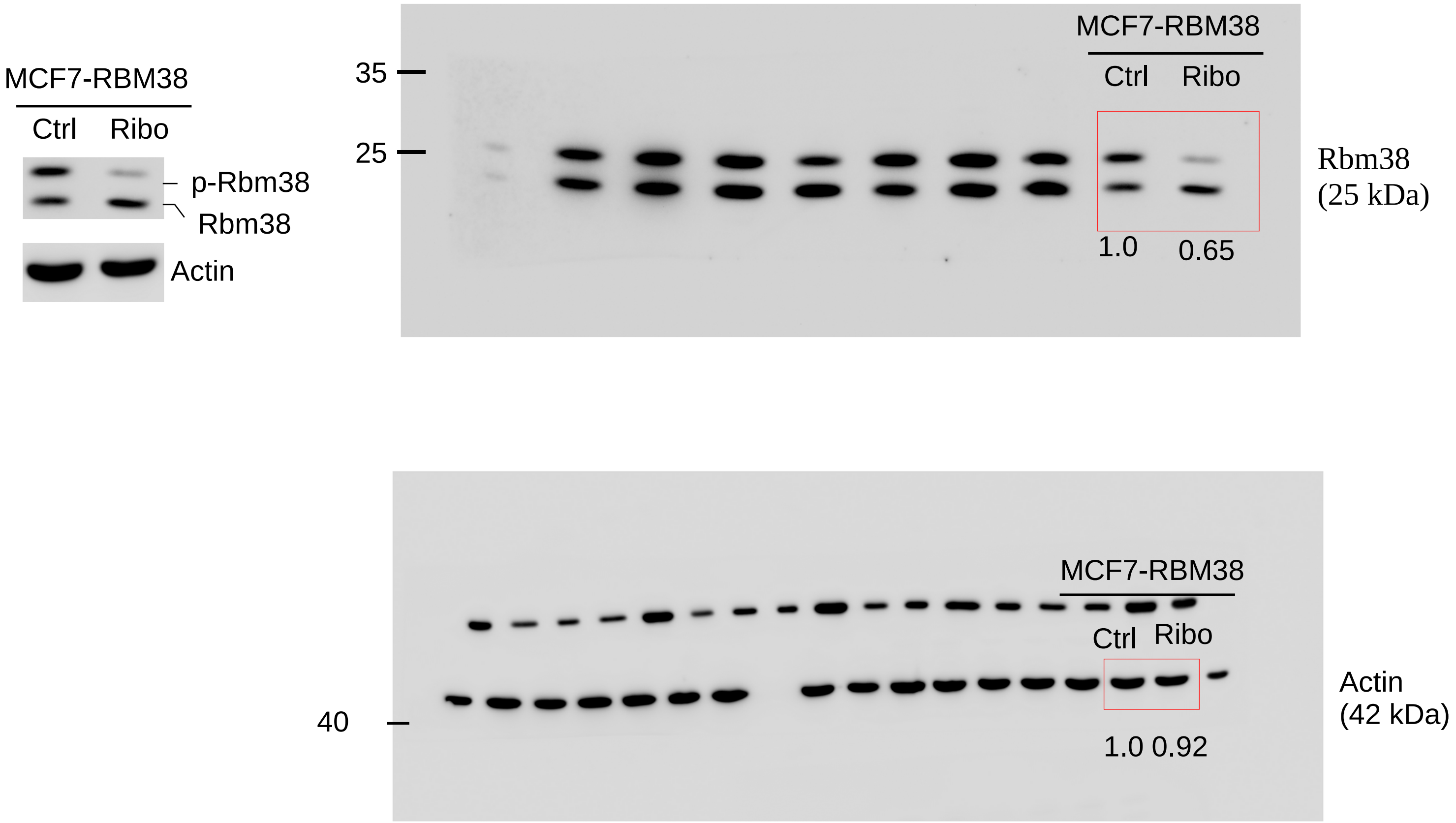

**Figure 4C**

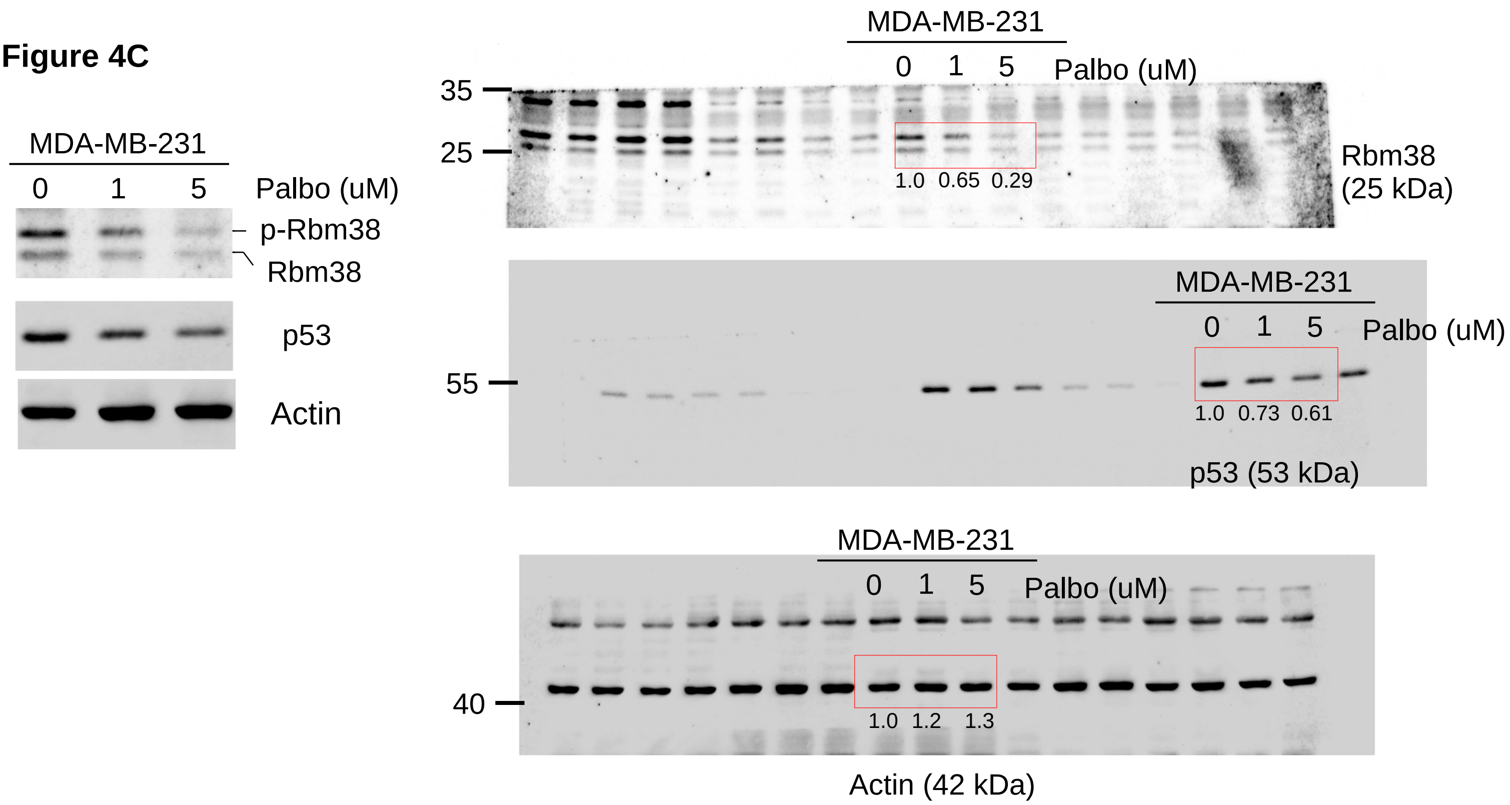


**Figure 4D**

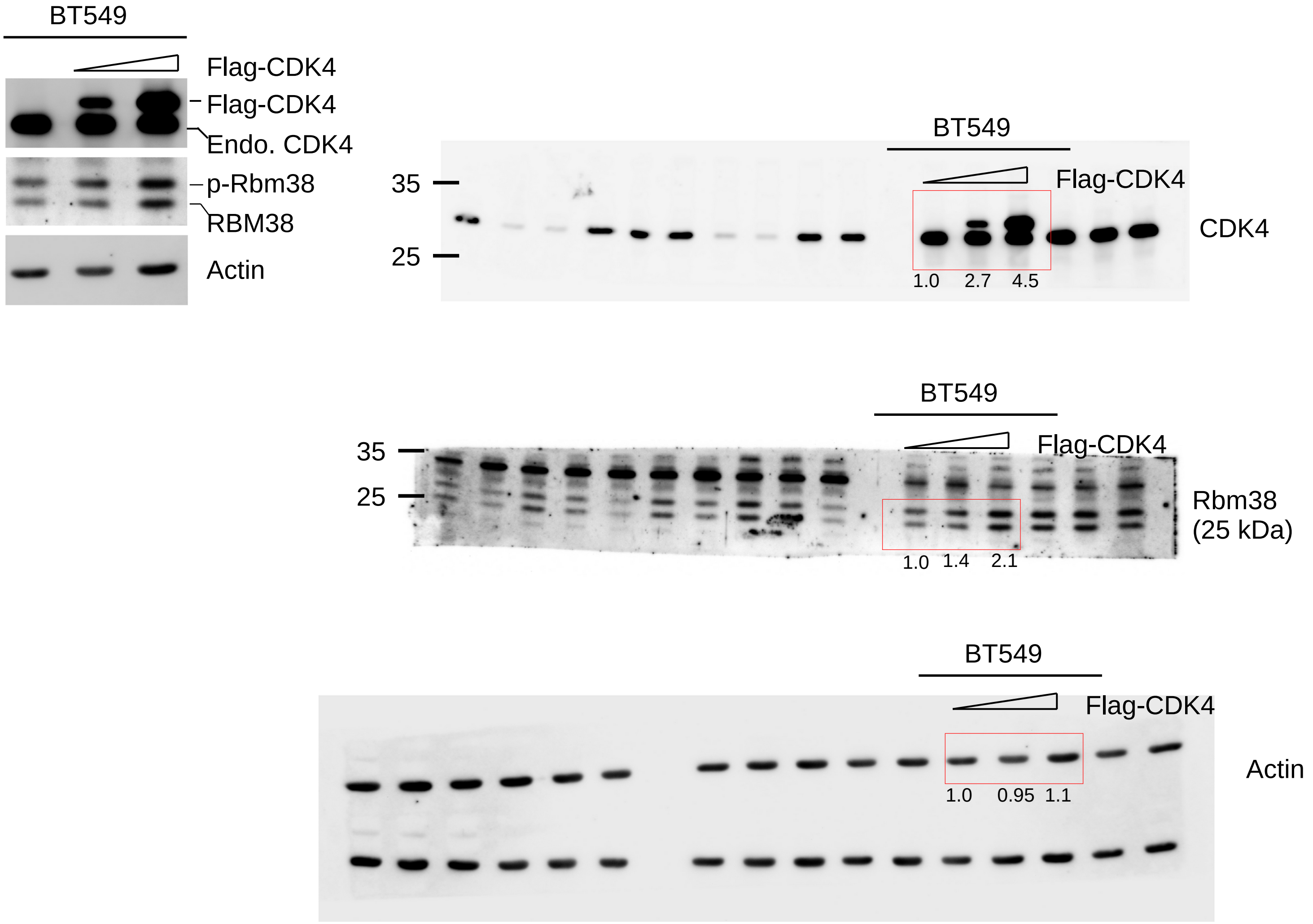

**Figure 4E**

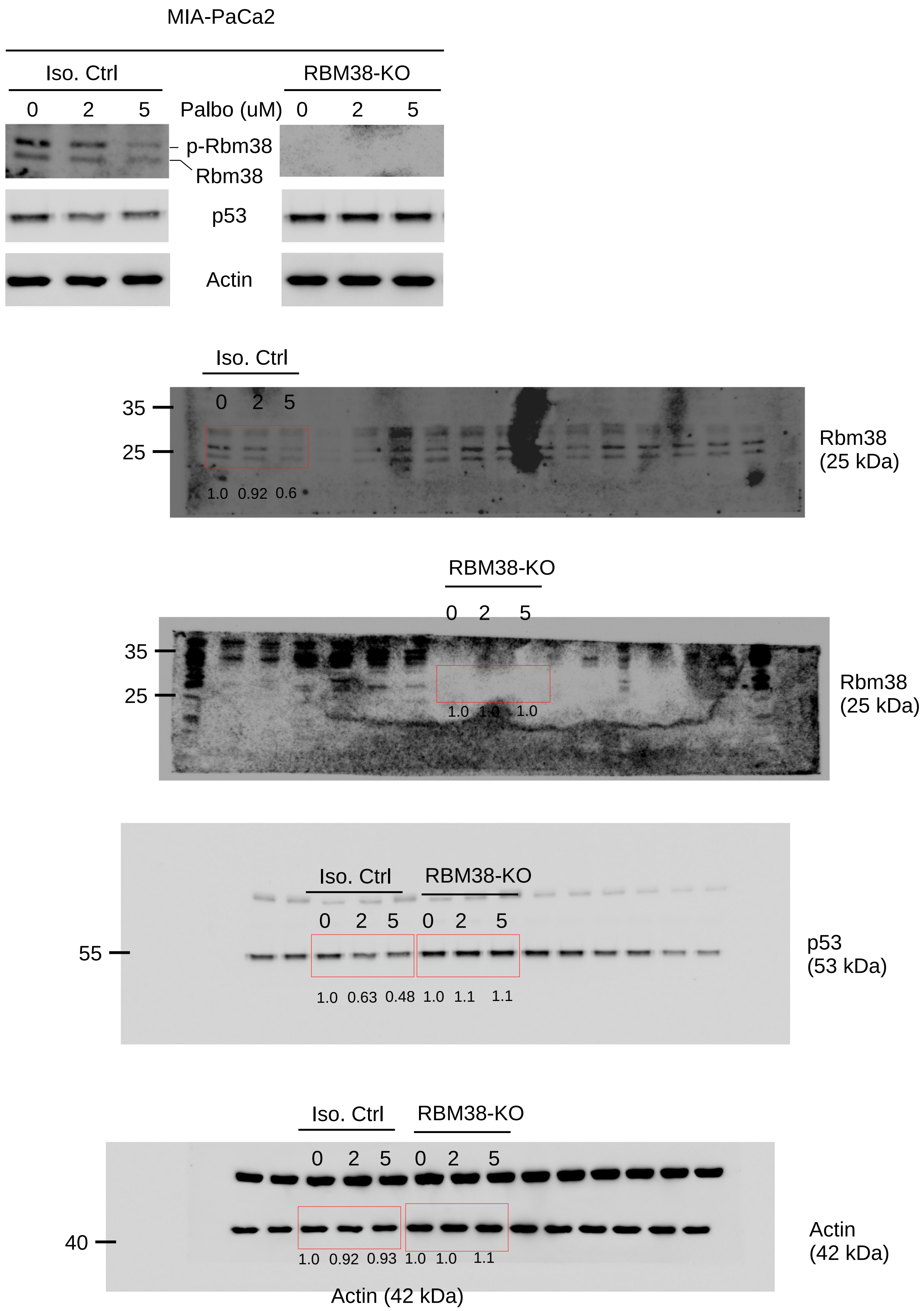

MIA-PaCa2
Iso. Ctrl
RBM38-KO
0 2 5
Palbo (uM)
0 2 5
p-Rbm38
Rbm38
p53
Actin
Iso. Ctrl
0 2 5
35
25
1.0 0.92 0.6
Rbm38 (25 kDa)
RBM38-KO
0 2 5
35
25
1.0 1.0 1.0
Rbm38 (25 kDa)
Iso. Ctrl
RBM38-KO
0 2 5 0 2 5
55
1.0 0.63 0.48 1.0 1.1 1.1
p53 (53 kDa)
Iso. Ctrl
RBM38-KO
0 2 5 0 2 5
40
1.0 0.92 0.93 1.0 1.0 1.1
Actin (42 kDa)
Actin (42 kDa)

**Figure 4F**

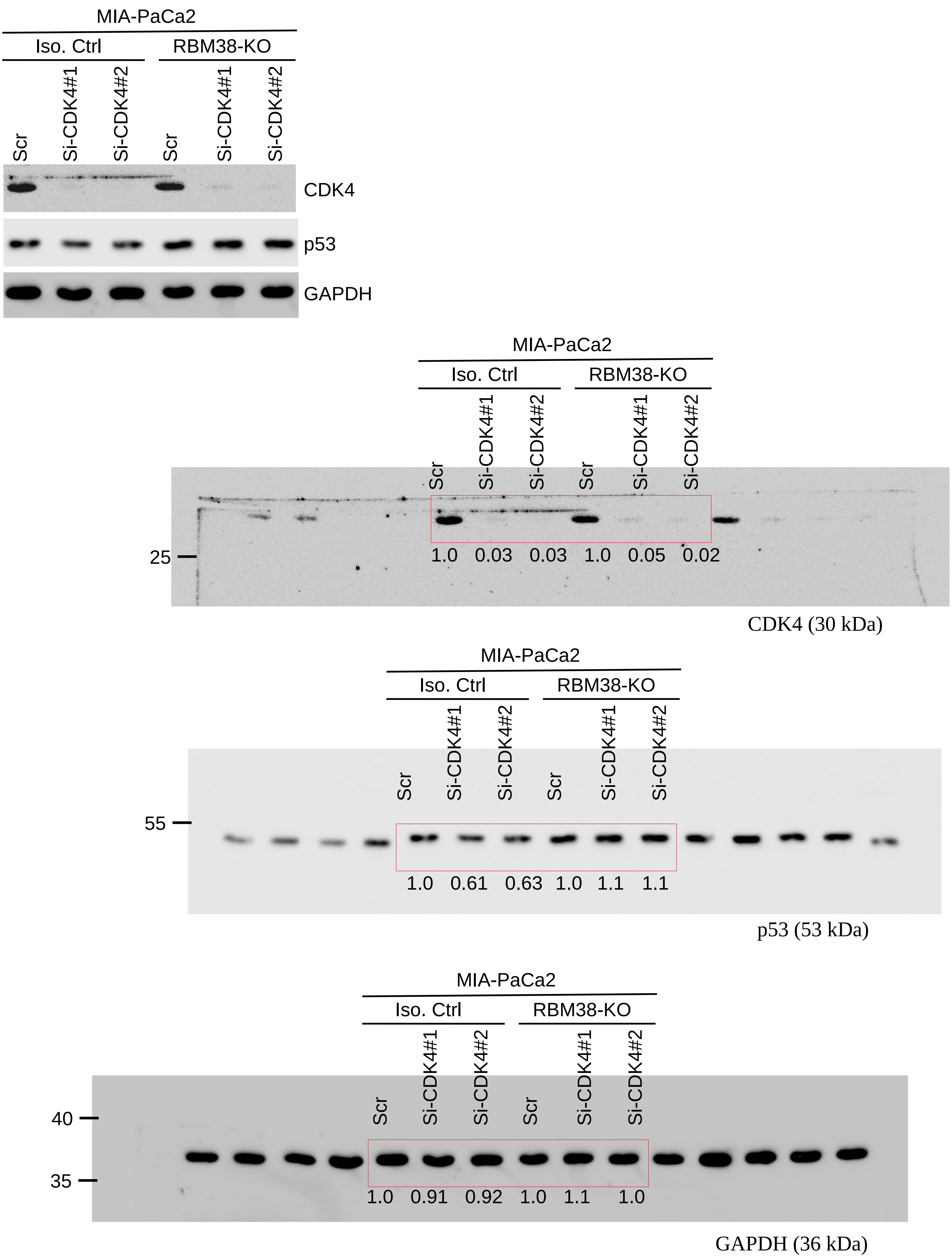
MIA-PaCa2
Iso. Ctrl
RBM38-KO
Scr
Si-CDK4#1
Si-CDK4#2
Scr
Si-CDK4#1
Si-CDK4#2
CDK4
p53
GAPDH
MIA-PaCa2
Iso. Ctrl
RBM38-KO
Scr
Si-CDK4#1
Si-CDK4#2
Scr
Si-CDK4#1
Si-CDK4#2
25
1.0 0.03 0.03 1.0 0.05 0.02
CDK4 (30 kDa)
MIA-PaCa2
Iso. Ctrl
RBM38-KO
Scr
Si-CDK4#1
Si-CDK4#2
Scr
Si-CDK4#1
Si-CDK4#2
55
1.0 0.61 0.63 1.0 1.1 1.1
p53 (53 kDa)
MIA-PaCa2
Iso. Ctrl
RBM38-KO
Scr
Si-CDK4#1
Si-CDK4#2
Scr
Si-CDK4#1
Si-CDK4#2
40
35
1.0 0.91 0.92 1.0 1.1 1.0
GAPDH (36 kDa)

**Figure 5A**

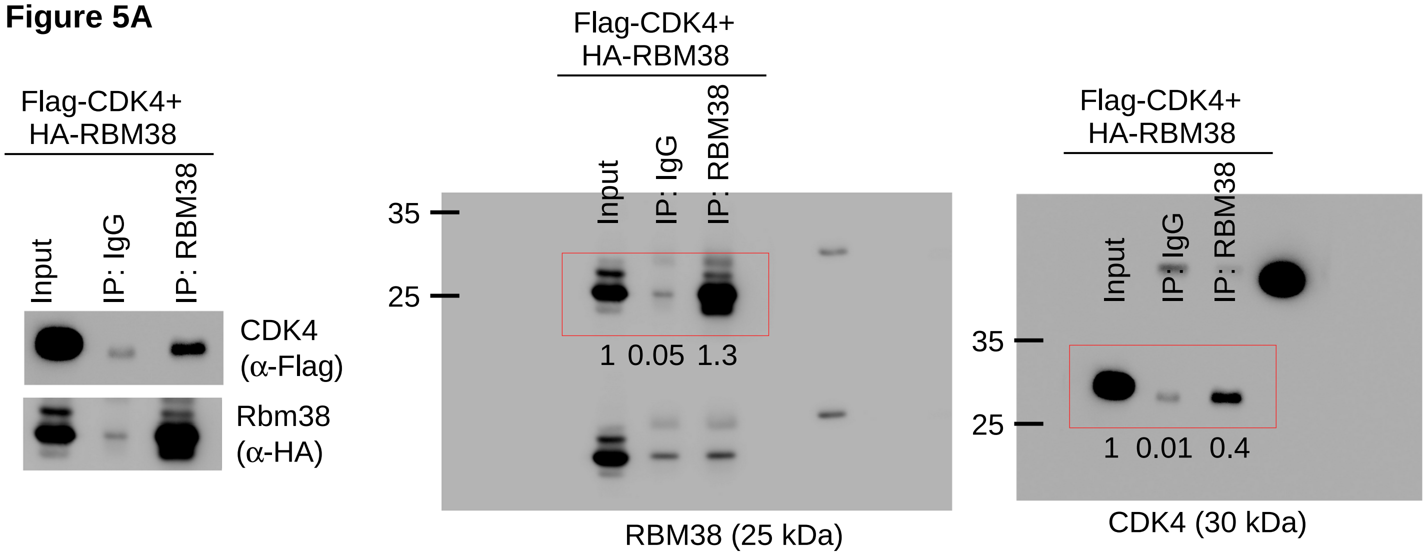


**Figure 5B**

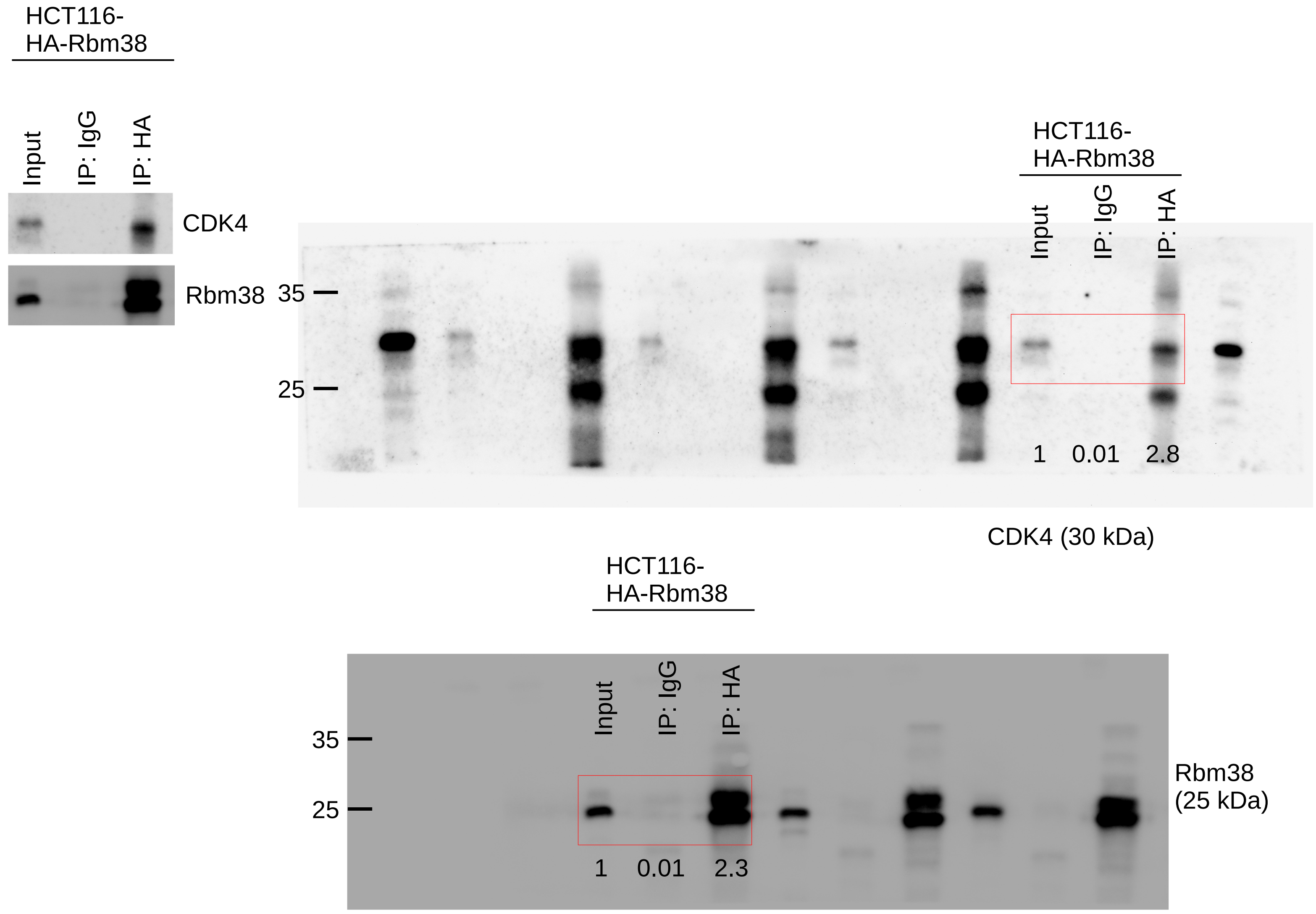

**Figure 5C**

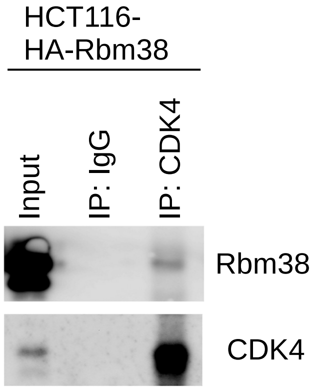
HCT116-
HA-Rbm38
Input
IP: IgG
IP: CDK4
Rbm38
CDK4

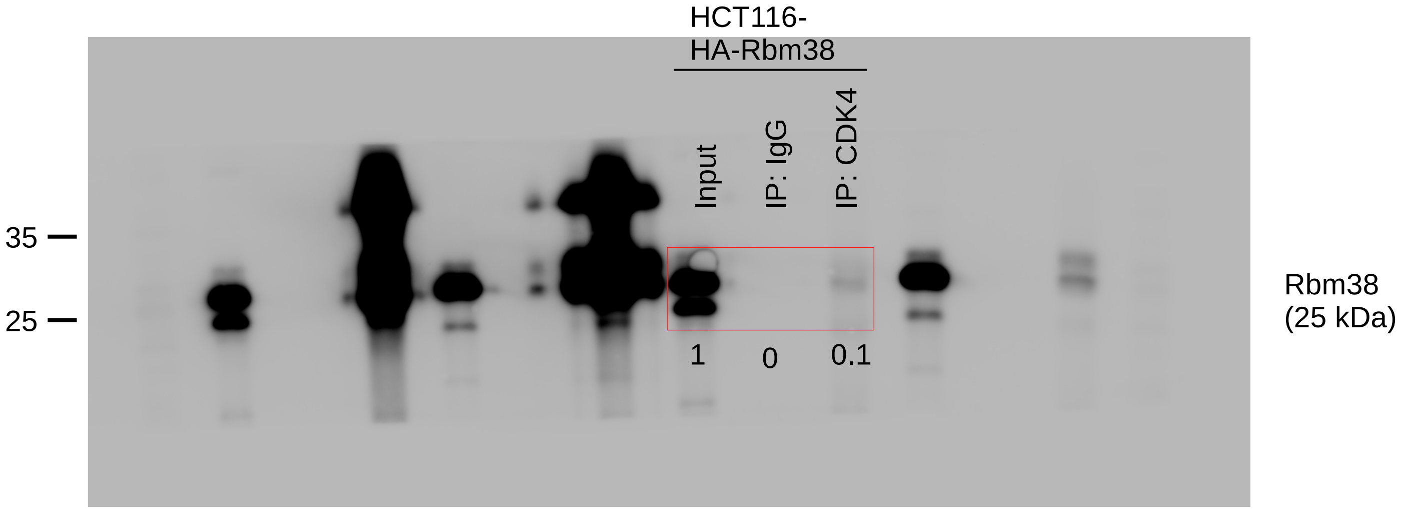
HCT116-
HA-Rbm38
Input
IP: IgG
IP: CDK4
35
25
1
0
0.1
Rbm38
(25 kDa)

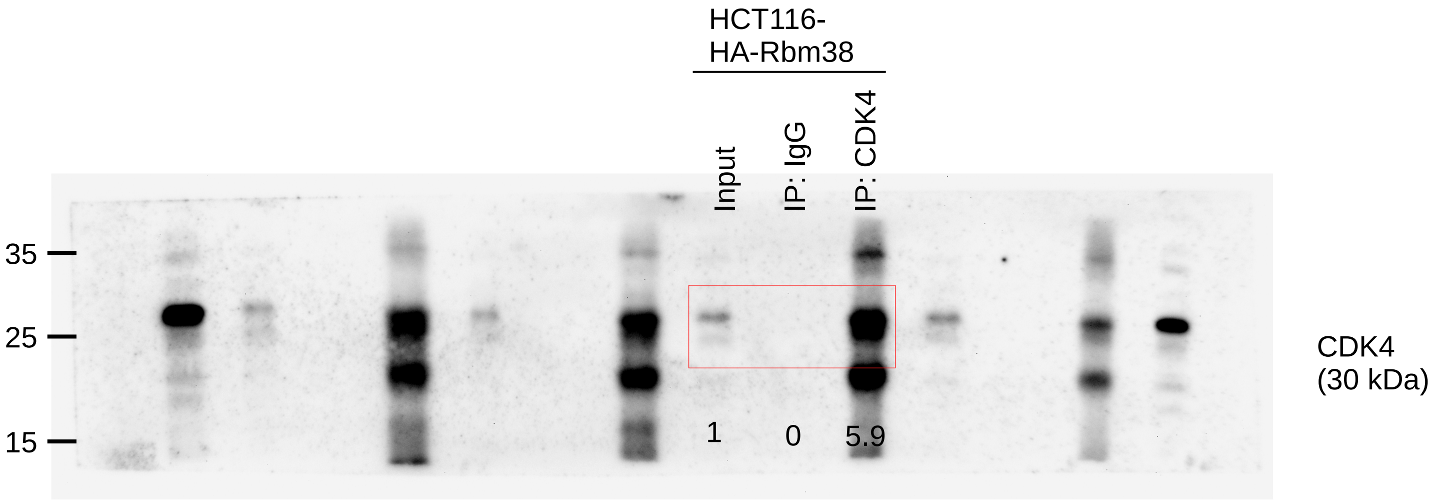
HCT116-
HA-Rbm38
Input
IP: IgG
IP: CDK4
35
25
15
1
0
5.9
CDK4
(30 kDa)

**Figure 5E**

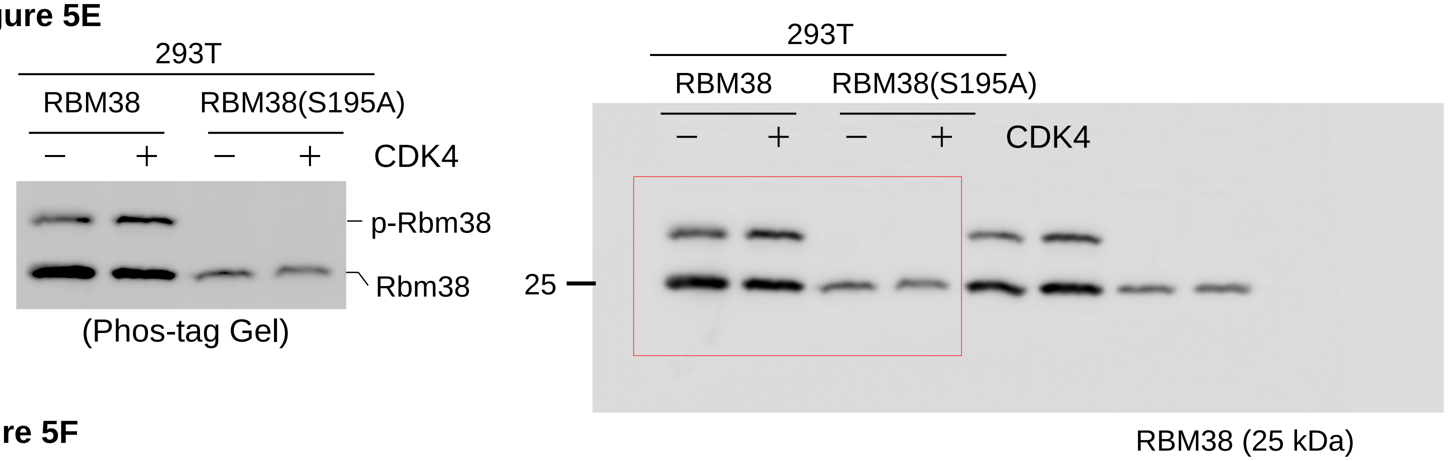


**Figure 5F**

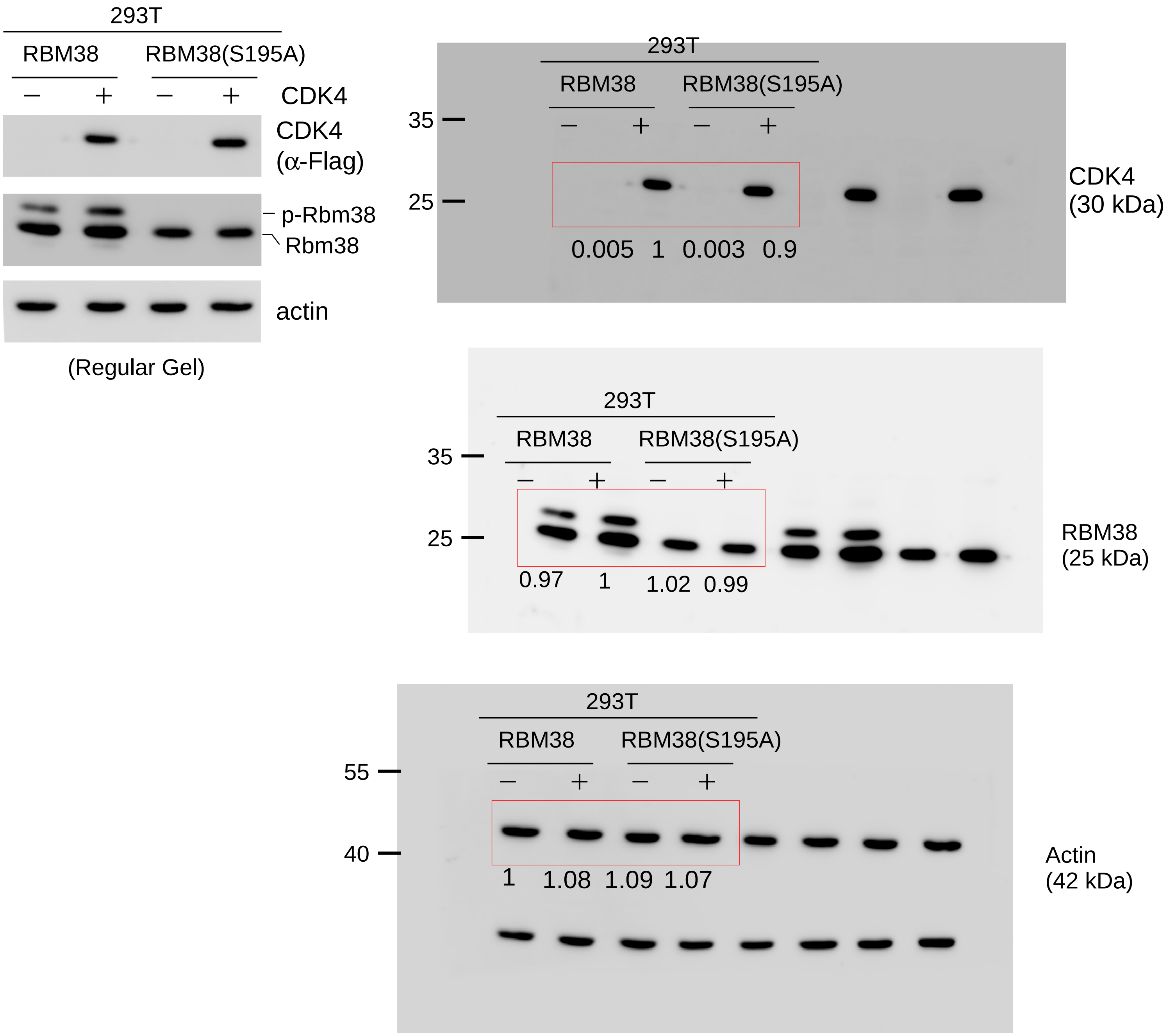

**Supplemental Figure 1A**

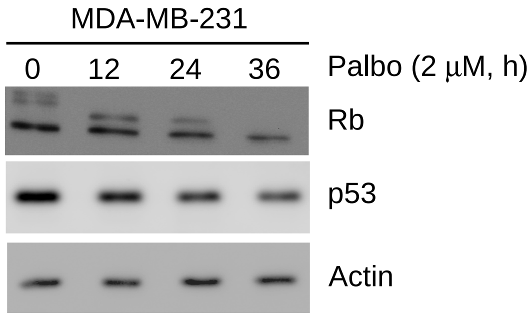


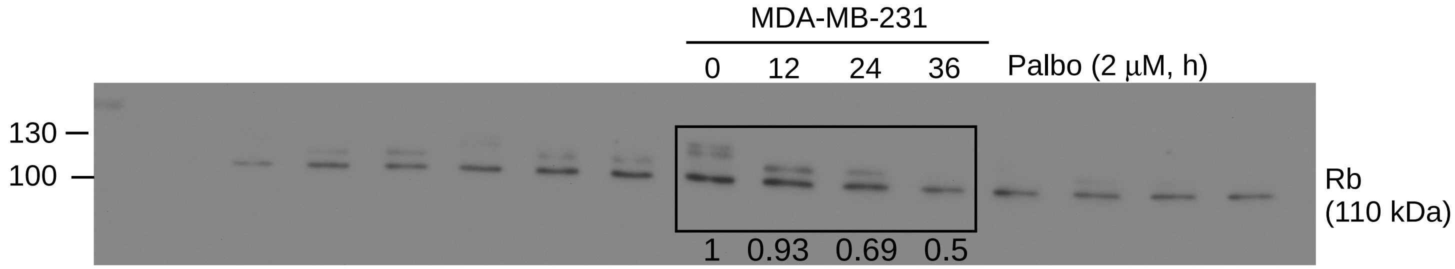


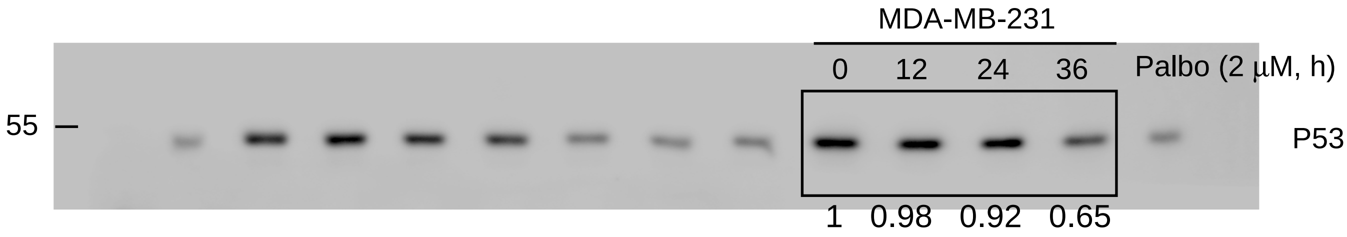


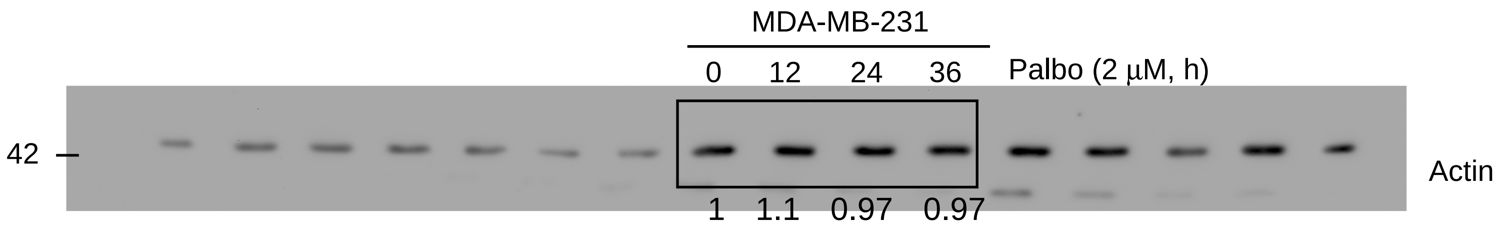

**Supplemental Figure 1B**

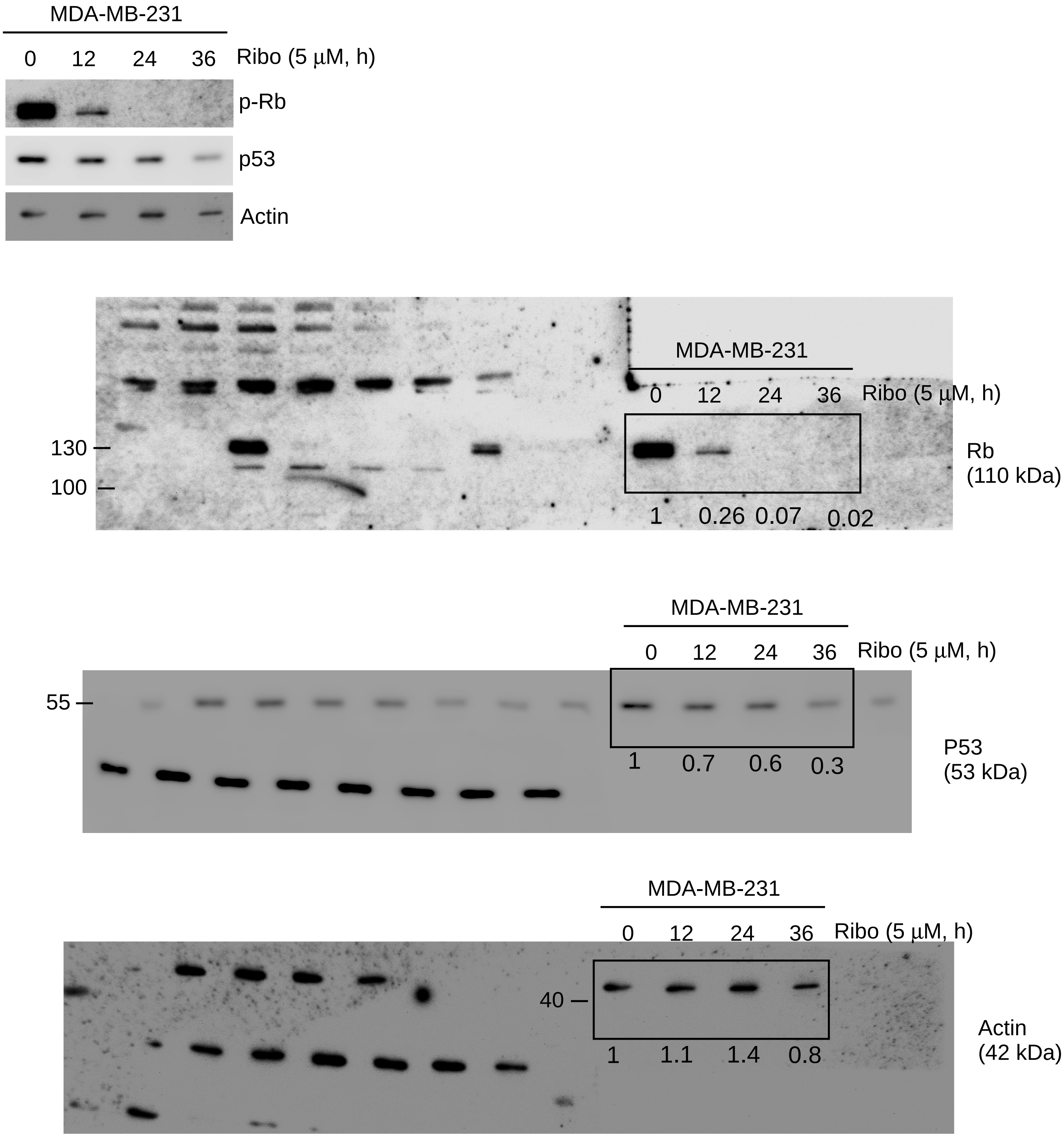

## Supplemental Figure 1C

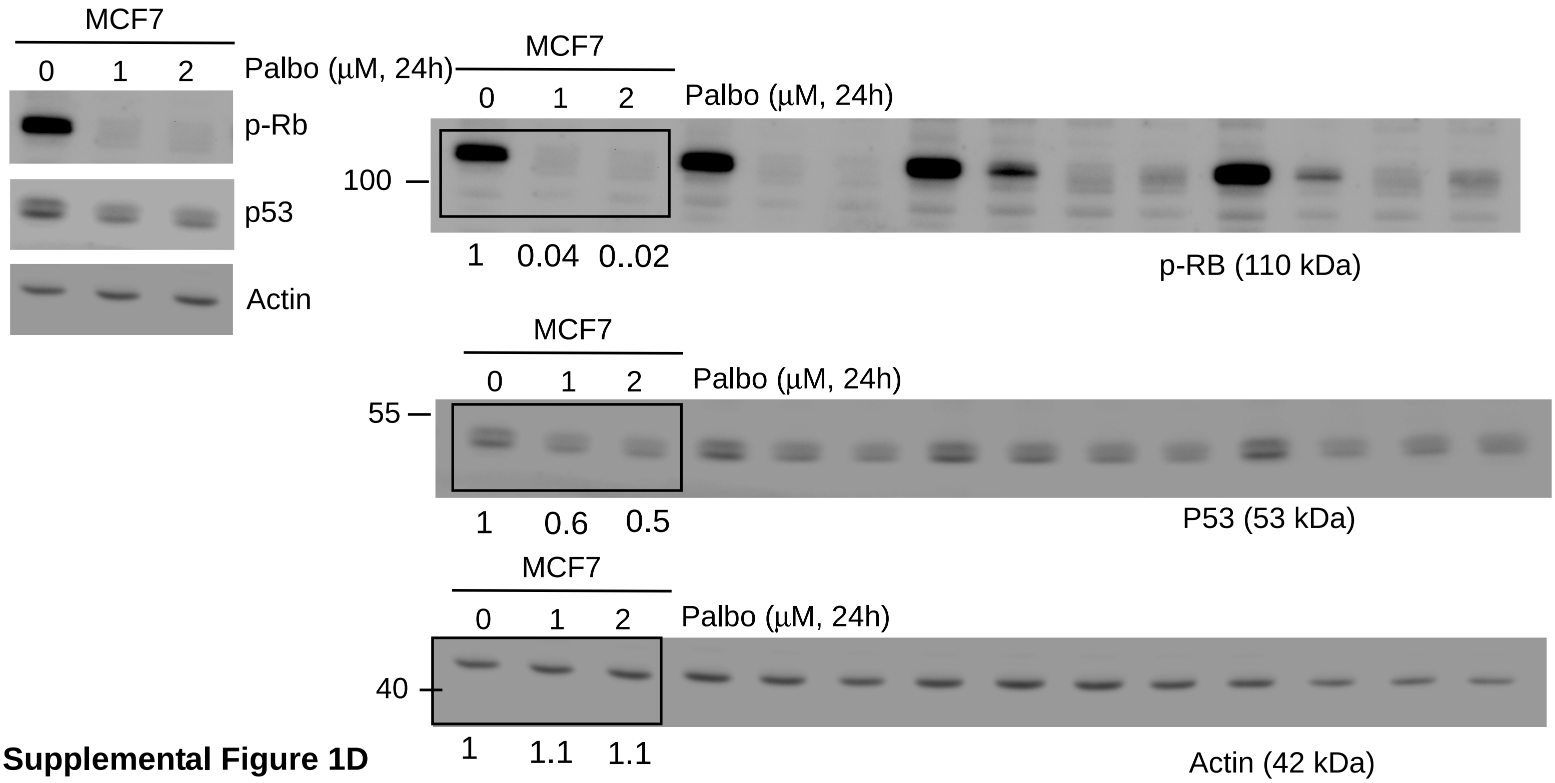


## Supplemental Figure 1D

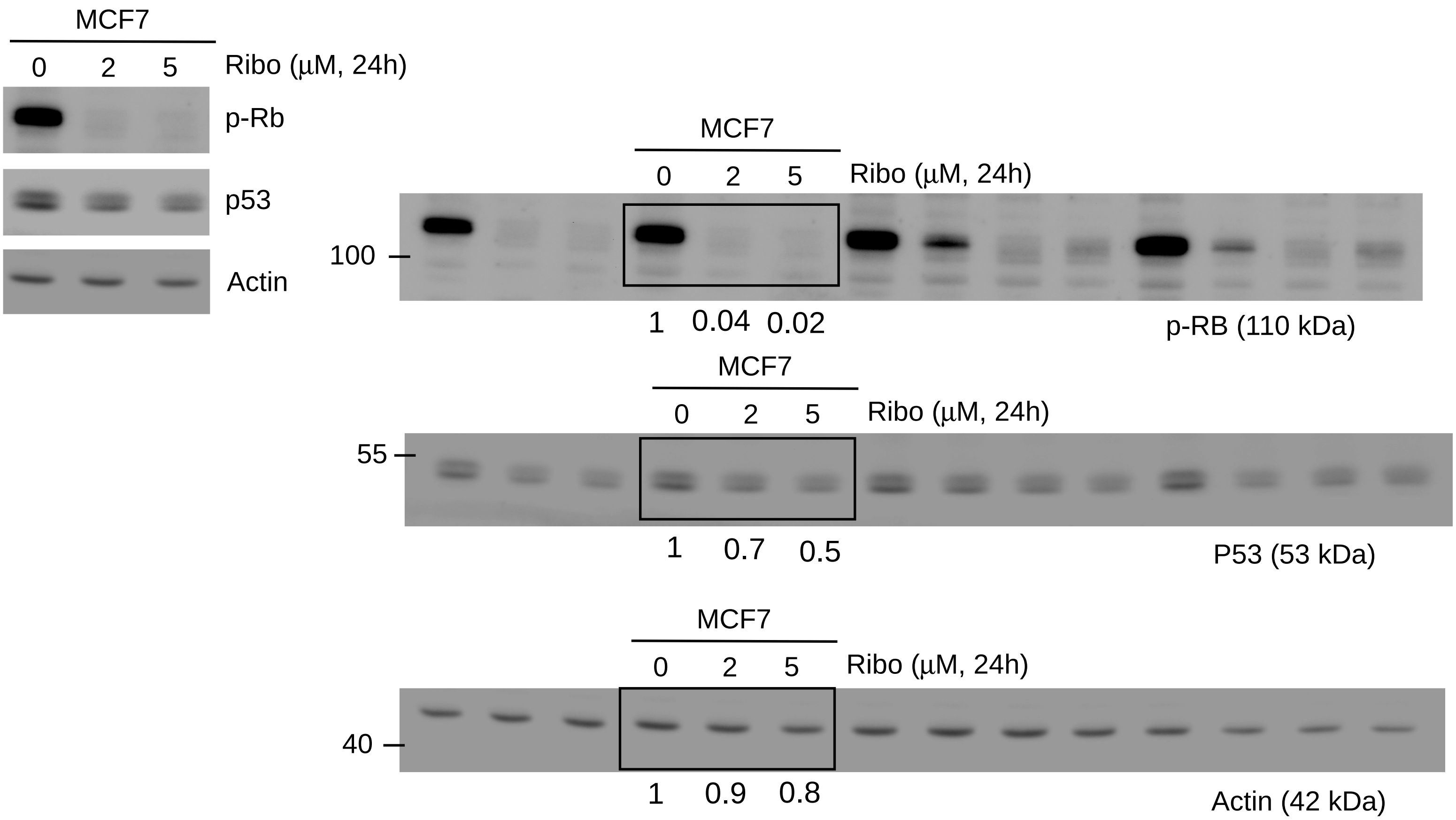

Supplement: Supplementary file 1 [file cancers-17-03339-s001.zip › cancers-3927674-supplementary materials/cancers-3927674-supplementary file.pdf]
